# Patients With LR-HPV Infection Have a Distinct Vaginal Microbiota in Comparison With Healthy Controls

**DOI:** 10.3389/fcimb.2019.00294

**Published:** 2019-08-28

**Authors:** Yunying Zhou, Lu Wang, Fengyan Pei, Mingyu Ji, Fang Zhang, Yingshuo Sun, Qianqian Zhao, Yatian Hong, Xiao Wang, Juanjuan Tian, Yunshan Wang

**Affiliations:** ^1^Medical Research & Laboratory Diagnostic Center, Jinan Central Hospital Affiliated to Shandong University, Jinan, China; ^2^Medical Research & Laboratory Diagnostic Center, Jinan Central Hospital, Shandong First Medical University & Shandong Academy of Medical Sciences, Jinan, China; ^3^Shandong LaiBo Biotechnology Co., Ltd., Jinan, China

**Keywords:** low-risk HPV, vaginal microbiome, 16S RNA sequencing, condyloma acuminatum, sexually transmitted infections

## Abstract

Condyloma acuminatum (CA) is a benign epithelium hyperplasia mainly caused by human papillomavirus (HPV), which is now the second most common viral sexually transmitted infection (STI) in China. In total, 90% of CA patients are caused by the low-risk HPV 6 and 11. Aside from low-risk HPV infection there are likely other factors within the local microenvironment that contribute to CA and there has been related research before. In this study, 62 vaginal specimens were analyzed using 16S rRNA gene sequencing. The diversity of the vaginal microbiota was higher and the composition was different with LR-HPV infection. While the relative abundance of dominant Firmicutes was lower, Actinobacteria, Proteobacteria, and Fusobacteria phyla were significantly higher; at the genus level Gardnerella, Bifidobacterium, Sneathia, Hydrogenophilus, Burkholderia, and Atopobium were higher. This study firstly confirmed a more accurate and comprehensive understanding of the relationship between low-risk HPV infection and vaginal microbiota, in order to provide a theoretical basis for further research on the occurrence and development of CA.

## Introduction

Condyloma acuminatum (CA) is a benign epithelium hyperplasia mainly caused by human papillomavirus (HPV), which is now the second most common viral sexually transmitted infection (STI) in China (Cong et al., 2016). The World Health Organization reports that CA is a widespread STI in the world with 101 million people infected every year, and the incidence rate can reach to 0.5~1% with an increasing trend year by year (Patel et al., 2013). In developing countries, the CA infection rate ranges from 4.5 to 22%, even as high as 80% in patients with cervical intraepithelial lesions (CIN), 23% in gynecological clinics and 19.9% in STI clinics. According to statistics, 10% of people in the United States are infected with CA. It is listed as one of the national priorities for prevention because it is closely related to cervical cancer and penile cancer (Vandepapeliere et al., 2005), but not all patients with CA develop cancer. Development of CA into cancer may be related to immune function, recurrence, skin damage or CA size (zur Hausen, 1990; Hagensee et al., 2004; Shilyansky et al., 2010; Pasmant et al., 2012; Stanley, 2012; Kumar et al., 2017). Up to now, more than 200 types of HPV have been found (Bernard et al., 2010), of which more than 40 types can be transmitted through direct sexual contact (Chesson et al., 2014). According to the probability of inducing reproductive tract malignant tumors, HPV clinical subtypes can be divided into low-risk (LR) and high-risk (HR) types, the former being the non-cancer-related type and the latter being the cancer-related type (Munagala et al., 2009). The incidence of HPV with low risk is generally higher than that of HR HPV, and it is also more infectious than high-risk transmission. Among HPV infected people, 80% of patients under the age of 50 were infected with condyloma acuminatum, and 2% infected patients had CA (Koutsky, 1997).

Studies have found that different genotypes of HPV have different degrees of damage to human reproductive organs after infection, so the prognosis of patients is often different (Sand and Thomsen, 2017). Although at least 40 HPV genotypes are related to CA like HPV 6, 11, 16, 18, 30, 31, 42, 43, etc. (Lorincz et al., 2002), about 90% of CA cases were caused by the low-risk HPV 6 and 11, particularly the former (Brown et al., 1999; von Krogh et al., 2001; Suzuki et al., 2014; Huang et al., 2015; Chen et al., 2016). Studies also found that the low-risk type is the main infection type of CA in China at present (Suzuki et al., 2014), and the causal relationship between other types of HPV and CA need further study (Hawkins et al., 2013). CA patients infected with HPV 16 and 18 may still develop into cancer because HPV 16 and 18 infections can lead to cervical cancer and penile cancer (de Araújo et al., 2018; Joob and Wiwanitkti, 2019; Sakamoto et al., 2019). Although low-risk HPV infection cannot directly lead to cancer, CA patients are often accompanied with other sexually transmitted diseases (AIDS, syphilis, gonorrhea, chlamydia, and mycoplasma, genital herpes, etc.), which are important factors leading to the recurrence and a worse prognosis of CA. In addition, if CA has not been cured for a long time and recurred often, or because of special parts (such as the vaginal cervix) which are difficult to find, the cancer may also occur after a few years.

Once CA causes skin damage, self-healing is more difficult because humans do not have natural immunity for HPV (Kanodia et al., 2007; Stanley, 2012). In addition, the virus lurks in the body's epidermal cells and cannot be easily destroyed by the human immune system (Schlecht et al., 2001; Moreno et al., 2002; Fehrmann and Laimins, 2003; Lee et al., 2003). Therefore, CA is difficult to self-heal without treatment. Although there are many clinical methods for treating CA, many patients still have different degrees of refractory recurrence for unknown reasons. The low cellular immune function of the human body (Vandepapeliere et al., 2005) and the immune escape mechanism of the HPV virus may also be some of the causes of the occurrence and recurrence of CA (Wang et al., 2014). Some studies suggest that CA recurrence is related to the level of sex hormones in patients or subclinical infection (Wang et al., 2014). There is also research reporting that HPV infection is associated with the imbalance of vaginal microbiota (Łaniewski et al., 2019). The vagina is a unique and dynamic microecological system. When the type, quantity, distribution, and proportion of vaginal microbial population change significantly under the influence of external pathogenic factors, it may be manifested as an imbalance in the structure of microbial communities and further induce diseases (Gao et al., 2013; Gilliland et al., 2013; Kentley et al., 2017). There are not only symbiotic, but also antagonistic relationships among the vaginal microbiota. Besides, the genital tract is a relatively closed environment, with anaerobic bacteria representing the majority of vaginal microorganisms. CA generally have a high recurrence rate, so the micro-ecology of CA patients will be changed because of HPV infection, malnutrition and trauma during the repeated treatments (Martinez-Domenech et al., 2019; Song et al., 2019). Due to the influence of HPV infection, the physical, chemical, and biological barrier functions of the organism are damaged, and it is easier to infect other microorganisms (Liu et al., 2016). In addition, the infection of other microorganisms like Chlamydia trachomatis or Vaginosis-associated bacteria also makes the treatment of HPV more difficult and complicated (Karim et al., 2018; Romero-Morelos et al., 2019). So, it is essential to effectively prevent HPV transmission and infection. However, all potential biological mechanisms are yet to be identified, and whether vaginal dysbacteriosis is the cause or result of LR HPV infection requires further examination.

Therefore, an accurate understanding of the composition of vaginal microbiota will help to clarify the relationship between vaginal microbiota and the occurrence of CA. Traditional molecular biology techniques cannot fully reflect the structural information of bacterial communities in the whole microenvironment, with the possibility of important information going unnoticed. In this study, we enrolled a total of 62 subjects consisting of a negative control group (NC, *n* = 20), and an HPV-positive CA group (LR6, *n* = 21; LR11, *n* = 21). We sought to afford a more accurate and comprehensive understanding of the relationship between low-risk HPV infection and vaginal microbiota in order to provide a theoretical basis for further research on the occurrence and development of CA.

## Materials and Methods

### Patients

A total of 62 individuals who underwent physical examination and/or diagnosis and treatment in the Jinan Central Hospital Affiliated to Shandong University were enrolled in this study. The samples were divided into three groups according to the results of LR-HPV screening: ① HPV negative control (NC, *n* = 20) and ② two HPV low-risk groups (LR6, *n* = 21; LR11, *n* = 21). Exclusion criteria: were persons under the age of 20 and over the age of 50, patients with immunodeficiency diseases; and patients with systemic diseases.

### Sample Collection and DNA Extraction

Vaginal samples were collected with a sterile cotton swab and placed in sterile containers. Samples were immediately frozen and stored at −80°C until DNA extraction. Microbial DNA was extracted from each sample using the DNA extraction Kit (Qiagen, Hilden, Germany) following the manufacturer's instructions. The extracts were kept at −20°C.

### 16S rRNA Gene Amplification and IlluminaMiSeq PE250 Sequencing

The V3-V4 region of the bacteria 16S ribosomal RNA genes was amplified by PCR (95°C for 3 min, followed by 30 cycles at 98°C for 20 s, 58°C for 15 s, and 72°C for 20 s and a final extension at 72°C for 5 min) using primers 341F 5′-CCTACGGGRSGCAGCAG)-3′ and 806R 5′-GGACTACVVGGGTATCTAATC-3.′ The KAPA HiFi Hot Start Ready Mix PCR kit (Kapa Bio systems, Inc. /Roche, Basel, Switzerland) was used for high-fidelity amplification. PCR reactions were performed in a 30 μL mixture containing 15 μL of 2 × KAPA Library Amplification Ready Mix, 1 μL of each primer (10 μM), 50 ng of template DNA and ddH_2_O. Amplicons were extracted from 2% agarose gels and purified using the Axy Prep DNA Gel Extraction Kit (Axygen Biosciences, Union City, CA, U.S.) according to the manufacturer's instructions and quantified using Qubit®2.0 (Invitrogen, U.S.). After preparation of the library, these tags were sequenced on a MiSeq platform (Illumina, Inc., CA, USA) for paired end reads of 250 bp, which were overlapped on their three ends for concatenation into original longer tags. DNA extraction, library construction and sequencing were conducted at Realbio Genomics Institute (Shanghai, China).

### Process of Sequencing Data

PANDA seq was used to assemble overlapping paired-end reads (Masella et al., 2012). Tags, trimmed of barcodes and primers, were further checked on their rest lengths and average base quality. 16S tags were restricted between 220 and 500 bp such that the average Phred score of bases was no worse than 20 (Q20) and no more than 3 ambiguous N. The copy number of tags was enumerated and the redundancy of repeated tags was removed. Only the tags with a frequency > 1, which tend to be more reliable, were clustered into OTUs, each of which had a representative tag. Operational Taxonomic Units (OTUs) were clustered with 97% similarity using UPARSE (http://drive5.com/uparse/), and chimeric sequences were identified and removed using Userach (version 7.0; Edgar, 2013). To minimize the deviation caused by the size difference, random leveling is performed for all samples with a subsample of 29,623 reads when there is enough sequencing depth. The assignment of bacterial taxonomy was performed against the RDP (Ribosomal Database Project) database with the RDP Classifier (http://rdp.cme.msu.edu) using a confidence threshold of 0.8 (Wang et al., 2007; Wang and Qian, 2009; Segata et al., 2011b; Cole et al., 2014). Rarefaction curves were generated with QIIME to test the current sequencing depth. Shannon and Simpson indices were used to estimate the α diversity (Kemp and Aller, 2004), which represented the abundance of species in a single sample, and were compared between groups with the Wilcoxon and Kruskal-Wallis rank sum tests using R3.1.0. The communities were compared based on phylogenetic distances using the weighted UniFrac metric to represent β diversity. Principal coordinates analysis (PCoA) was performed on the resulting matrix of distances between each pair of samples. A heatmap was also employed. Based on the UniFrac phylogenetic distances, a significance test for the clustering of samples was carried out using one-way analysis of similarities (ANOSIM). LEfSe analysis was applied to identify the different abundant bacterial taxa among the groups (Segata et al., 2011a,b). Only those taxa that obtained a log-linear discriminant analysis (LDA) score > 2 were ultimately considered (Segata et al., 2011b; Masella et al., 2012).

### Statistical Analysis

In addition to the statistical analyses described above, the statistical analysis of quantitative data was conducted by Student's *t*-test using SPSS 16.0 (IBM, Armonk, NY, USA). A *P*-value of < 0.05 was considered statistically significant.

## Results

### Increased Diversity of Genital Tract Microbiota in HPV Low-Risk Group

**Figure 1:**
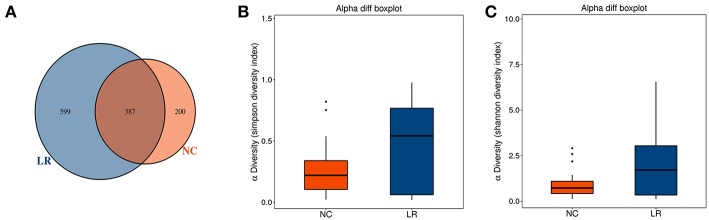
Increased diversity of genital tract microbiota in HPV low-risk group. **(A)** Venn analysis of bacterial operational taxonomic units' (OTUs) composition between negative control samples (NC) and positive group (LR6-11). **(B,C)** Shannon diversity and Simpson diversity representing community richness were calculated.

A total of 62 genital tract samples were obtained from the study population for sequencing (Table 1). After processing with PANDA seq, 3 272 633 high quality reads with a mean of 54,543 reads per sample (the standard deviation is 9,913) were obtained. In order to facilitate the analysis of downstream species diversity, clean reads were clustered into operational taxonomic units. According to the abundance of OTU in each sample, the common and unique OTU between each sample or group is calculated (Kemp and Aller, 2004) and each OTU is considered to represent one species. A total of 587 OTUs were identified in the NC group while 986 OTUs were identified in the LR group. By comparing the OTUs between the two groups, we found that 387 core OTUs were present in the two groups. Two hundred OTUs were uniquely identified in the healthy cluster, and 599 OTUs were identified in the positive patients (Figure 1A). In order to distinguish the species diversity in a single sample, we used Analysis of Single Sample Alpha diversity and found that microbial diversity was significantly increased in LR-HPV infectors, as calculated by the Shannon and Simpson diversity indices (Figures 1B,C).

**Table 1:** Characteristics of the study subjects.

**Characteristics**	**LR group (*n* = 42)**	**NC group (*n* = 42)**	***P*-value**
Age (years)^a^	32 (4.1)	34.3 (4.1)	0.772
BMI (kg/m^2^)^a^	22.6 (2.4)	21.8 (1.9)	0.235
Single want^a^	14 (33.3%)	–	
Multiple wants^a^	28 (67.7%)	–	
Acetate white test^a^	+	–	
Colposcopy^a^	+	–	
HPV genotyping^a^	+	–	
BV	6 (14.2%)	–	
Other vaginosis	7 (16.6%)	–	

a *Data are shown as mean (SD). SD, standard deviation; BMI, body mass index; BV, bacterial vaginosis*.

### Analysis of Microbial Beta Diversity

**Figure 2:**
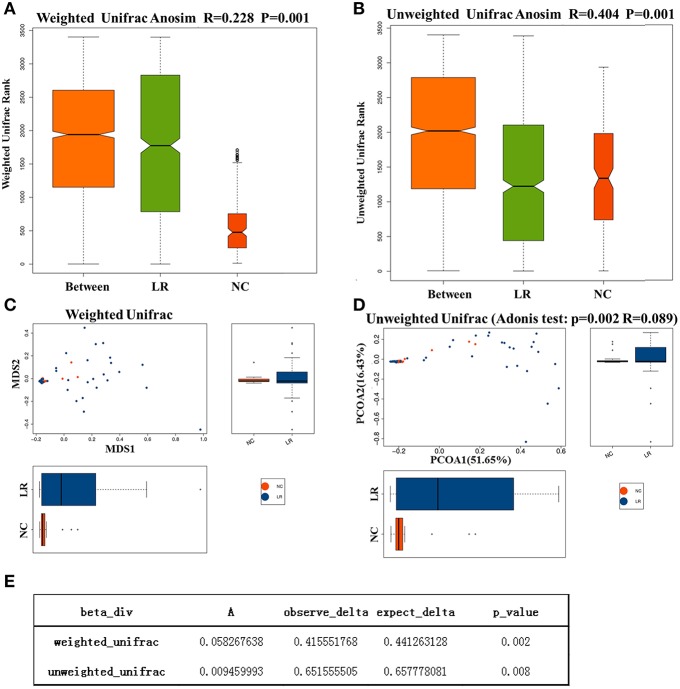
Analysis of microbial Beta diversity. **(A,B)** Difference between and within groups was assessed by one-way analysis of similarities (ANOSIM) analysis. **(C,D)** NMDS analysis and PCOA analysis between NC and HPV-LR groups. **(E)** MRPP analysis.

To investigate the different compositions of vaginal microbiota between groups, we calculated the UniFrac phylogenetic distances of the microbial composition for the samples. ANOSIM analysis is a non-parametric test used to determine whether a grouping is meaningful by examining whether the differences between groups are significantly greater than those within groups. Our results (*R* = 0.228, *p* = 0.001) indicate that the differences between groups were greater than those within groups with great statistical significance (Figures 2A,B). To further demonstrate the differences in species diversity between samples, the PCoA and NMDS (non-metric multidimensional scaling) methods were used (Mohd Shaufi et al., 2015; Xu et al., 2015; Yasuda et al., 2015). Figures 2C,D shows that proximal samples exhibit relatively similar species composition. As shown in the figure, the vaginal microbes in the above groups exhibit some similarities in flora composition, and they also have a tendency to separate from each other. The results of MRPP analysis, used to analyze the difference in microbial community composition between groups, indicated a significant difference between the NC and HPV-LR groups (*p* = 0.001, Figure 2E).

### Composition of Microbial Communities

**Figure 3:**
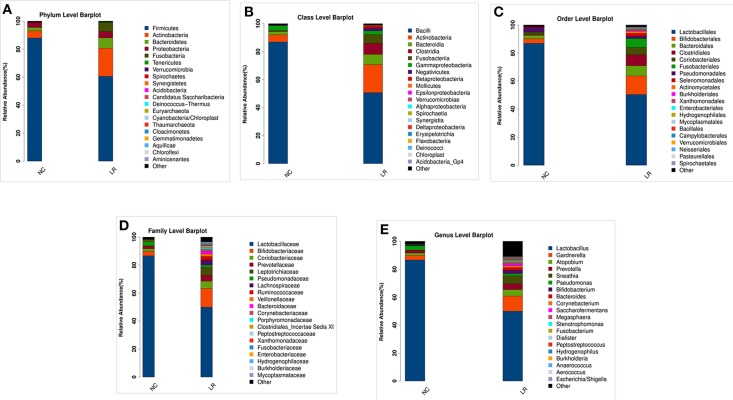
Composition of microbial communities. **(A)** The difference in the abundance of vaginal microbial communities at the Phylum level. **(B)** The difference in the abundance of vaginal microbial communities at the Class level. **(C)** The difference in the abundance of vaginal microbial communities at the Order level. **(D)** The difference in the abundance of vaginal microbial communities at the Family level. **(E)** The difference in the abundance of vaginal microbial communities at the Genus level.

According to the species annotation results, a corresponding histogram of species profiling was made for each group at the classification levels of phylum, class, order, family, and genus. The histogram showing the average relative abundance of species visualizes the species and proportion of each group with higher relative abundance at different classification levels (Figures 3A–E). The analysis of the composition and microbial abundance of the vaginal microbiota at the phylum and genus levels reveals the overall structure of vaginal microbial communities. On the whole, the composition of vaginal microbiota in each group is different at the phylum level (Figure 3A). In brief, sequence alignment analysis using the RDP database showed that 20 phyla were detected. In the NC group, the proportion of Firmicutes was the highest, accounting for 88%. Compared with the NC group, the content of Firmicutes was significantly lower in the LR group (down to 60%), while Antinobacteria and Fusobacteria were 4 and 111 times more than that in NC group. In addition, Spirochaetes and Cloacimonetes only existed in the HPV-LR group. We further analyzed the characteristics and changes in vaginal microbial community structure in each group at the genus level (Figure 3E and Table 2). *Lactobacillus* was the dominant bacterium in the NC group (accounting for 85.59%), which also included *Gardnerella, Prevotella, Pseudomonas, Atopobium*, and other bacteria. The community structure of the vaginal microbiota in the HPV-LR group was significantly altered at the genus level, with the most prominent manifestation being the decrease in *Lactobacillus* content, the increase of *Gardnerella* (up to 3 times), *Bifidobacterium* (up to 146 times), *Sneathia* (up to 110 times), *Hydrogenophilus* (up to 3,264 times), *Burkholderia* (up to 177 times), and *Fusobacterium* (up to 122 times), which together constituted the dominant bacteria of the vaginal microbiota with decreased *Lactobacillus*. Figure 3 shows the average relative abundance of dominant microorganisms in samples from the NC and LR groups, including analysis of significant differences and non-significant differences.

**Table 2:** Abundance of vaginal microbial communities at the genus level.

**Tax_name**	**NC**	**LR**	**LR/NC**
Lactobacillus	86.59268	49.95242	0.576867
Gardnerella	3.260136	10.82894	3.321623
Sneathia	0.051649	5.691845	110.2023
Atopobium	1.701381	4.615863	2.71301
Prevotella	2.011613	4.17388	2.074893
Pseudomonas	3.227391	1.569485	0.486302
Bifidobacterium	0.016541	2.429097	146.8513
Bacteroides	0.127097	1.890423	14.87384
Corynebacterium	0.014516	1.328762	91.53931
Saccharofermentans	0.000844	1.331092	1577.238
Megasphaera	0.163049	1.116491	6.84758
Fusobacterium	0.005401	0.660442	122.2768
Stenotrophomonas	0.165243	0.565037	3.419427
Peptostreptococcus	0.00692	0.619853	89.57027
Hydrogenophilus	0.000169	0.551052	3264.762
Dialister	0.242379	0.421085	1.737299
Burkholderia	0.002701	0.478714	177.2619
Aerococcus	0.184485	0.270703	1.467346
Anaerococcus	0.228707	0.245064	1.071516
Escherichia/Shigella	0.043041	0.301487	7.004669
Other	1.954056	10.95826	5.607958

### Bacterial Taxa Differences Between Healthy Controls and HPV-LR Samples

**Figure 4:**
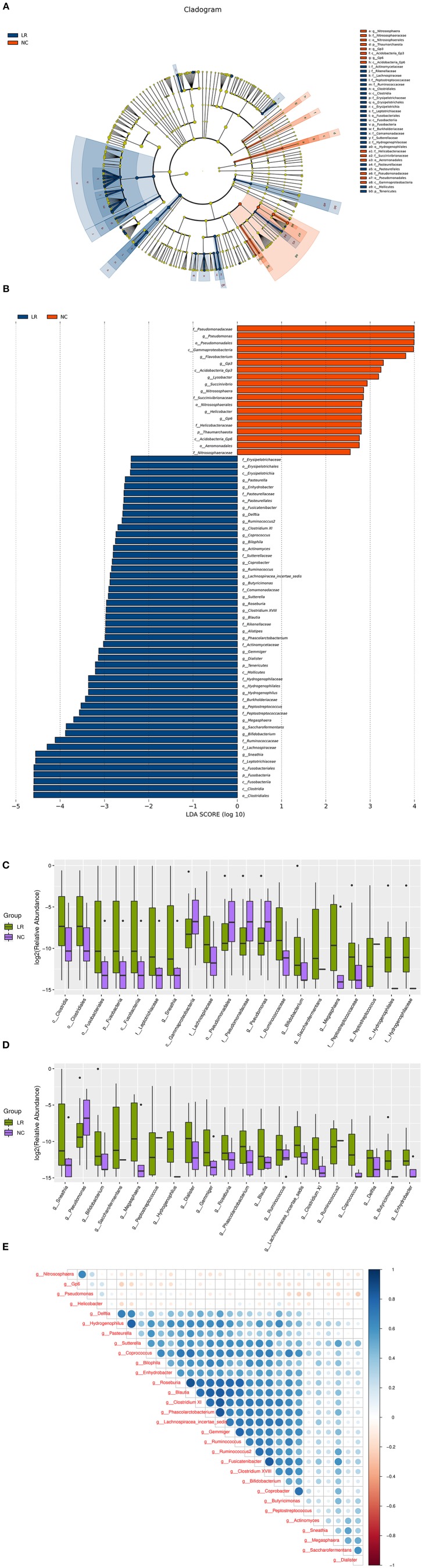
Bacterial taxa differences between healthy controls and HPV-LR positive samples. **(A)** Cladogram showing the most differentially abundant taxa identified by LEfSe. Red indicates clades enriched in the NC group, whereas blue indicate clades enriched in the HPV-LR group. **(B)** Comparisons of vaginal bacteria between the NC and HPV-LR groups. **(C)** Boxplots with relative abundance of the different OTUs. **(D)** Boxplots with relative abundance of the different microbial taxa at the general level. **(E)** Spearman correlation coefficient analysis of dominant species.

To further investigate the differences in the abundance among groups and to explore macrogenomic biomarkers at the metagenomic level, LefSe analysis was performed (the threshold is *P* < 0.05, the linear discriminant analysis LDA value > 2). We found 69 differentially abundant taxa (19 taxa were higher in the NC group and 50 taxa were higher in the HPV-LR group) among the two groups (Figures 4A,B). The most significant difference among the two groups was that *Pseudomonas, Nitrososphaera, Gp6* and *Helicobacter* were mainly enriched in the NC group and *Actinobacteria, Bifidobacterium, Actinomyces, Brucellaceae*, and *Hydrogenophaga* were mainly enriched in the HPV-LR group. Next, boxplot diagrams were used to display the differences visually (Figures 4C,D). In order to identify important patterns and relationships among the dominant species, we selected the 30 most abundant species and drew a Spearman correlation heatmap among the dominant species using the CORRPLOT package of R software. Figure 4E shows that *Pseudomonas, Nitrososphaera*, Gp6, and *Helicobacter* exhibited a negative correlation compared with all other dominant species. We further analyzed the microecology between low risk HPV subtypes 6 and 11, and found that there was no significant difference between them (the results in Supplemental Figure 1).

## Discussion

LR-HPV 6/11 is the main subtype which is closely related to the occurrence of condyloma acuminatum (CA), while partial CA can be transformed into VIN or squamous cell carcinoma and further lead to vaginal and cervical epithelium neoplasia and canceration by means of sexual transmission. In fact, most patients can clear HPV infection through their own immune response. The median clearance time for female cervical HPV infection is 9.4 months, while the clearance time for male genital HPV infection is 7.5 months. Within 1 year after diagnosis of CA, 80% of female patients can be cured after treatment (Vinay et al., 2009). However, there are also some patients who are unable to clear HPV infection due to various reasons, resulting in persistent infection. The pathogenesis of CA is still unclear, and research in recent years suggests that the occurrence of apoptosis may be an important reason for the recurrence of CA after cure and the development of cancer (Cokluk et al., 2015).

As an important part of the vaginal self-purification function and biological barrier, the resident flora and opportunistic pathogens of the vagina maintain the microecological balance of the vagina, and the acidic environment of the vagina can inhibit the proliferation of pathogenic bacteria. *Lactobacillus* (the dominant bacteria which colonized on the surface of vaginal mucosal epithelial cells) can decompose vaginal epithelial glycogen to produce lactic acid, maintain a weakly acidic environment in the vagina and help maintain the balance between normal vaginal microbiota and microecology (McMillan et al., 2011; Huang et al., 2014; Sgibnev and Kremleva, 2015). *Lactobacillus* can also play a role in inhibiting pathogen growth and enhancing anti-infection ability by producing various metabolites or stimulating immune cells to produce various cytokines (Matsuzaki, 1998; Aroutcheva et al., 2001; Boskey et al., 2001; Wang et al., 2017; Liu et al., 2019; Wu et al., 2019). If the balance is destroyed, it can easily lead to various pathogenic infections such as bacterial vaginosis (BV), vaginal pseudofilariasis (VVC), trichomonas vaginitis (TV), aerobic vaginitis (AV), etc. More studies have shown that there is a certain correlation between vaginal microbiota imbalance and diseases such as BV and HPV infection. The former can cause mucosal damage and increase the chance of HPV invasion. Conversely, HPV infection can change mucosal metabolism or immune response, further induce vaginal microorganism community structure imbalance and vaginal infection occurs; the two tend to form a vicious circle (Meng et al., 2016; Zhou et al., 2016). The vaginal microecological characteristics of BV are increased *Gardnerella* and anaerobic bacteria and decreased *Lactobacillus*, and it has been reported that BV is conducive to the persistence of HPV infection and may be one of the factors affecting the poor prognosis of CA (Song et al., 1999; Guo et al., 2012).

In this study, we analyzed the correlation of vaginal microbes between normal patients and CA patients infected with HPV LR-6/11 via Illumina high-throughput sequencing technology which has often been used in previous studies for HR-HPV (Shannon et al., 2017; Kwasniewski et al., 2018; Chao et al., 2019; Klein et al., 2019) and found that the diversity of the flora increased, the proportion of *Lactobacillus* was lower, various anaerobic bacteria such as *Gardnerella* and *Sneathia* were over-proliferated, *Brucella* and other rare species proliferated, there was excessive growth of various miscellaneous bacteria, and the composition of BV or other vaginally infections were more common in CA patients with LR HPV infection.

Most of the taxa identified by LEfSe had very low relative abundance (<1%), but many studies of human microecology have found that the changes in the abundance of many low-abundance species have an important influence on human health and disease progress. Lee suggested that anaerobic bacteria *Sneathia* is closely related to HPV infection and may even be considered a microbial marker of LR-HPV infection (Larsen et al., 2013). Our results showed that *Sneathia* (up to 110 times) was higher in CA patients with LR-HPV infection, which is consistent with the existing conjecture. Another kind of anaerobic bacterium *Bifidobacteria* may enhance the antitumor immunity and efficacy of immunotherapy (Liwen et al., 2018), which was also significantly higher (up to 146 times) in the LR-6/11 infection group. Moreover, the excessive growth of various miscellaneous bacteria and some conditional pathogens like *Atopobium, Hydrogenophaga, Burkholderia, Stenotrophomonas* were accompanied by the accumulation of a large number of harmful metabolites in the LR-6/11 infection group.

In addition, we found there are some inconsistencies between Figure 3 and LEfSe analysis. For example, *Lactobacillus* and *Gardnerella* appear to be higher in the LR group in Figure 3 but are not identified as differentially abundant by LEfSe. That is because LEfSe shows the species whose abundance varies significantly between the NC and LR groups (the threshold is *P* < 0.05, the linear discriminant analysis LDA value > 2). Due to the higher *P*-value of *Lactobacillus* and *Gardnerella*, they cannot be shown in the LEFSe diagram. We added Supplemental Data 2 to display the results of LEfSe's analysis (containing *P*-value for all species). Although the Wilcoxon rank-sum test is a commonly used method for LEfSe difference analysis in the study of flora, it may not apply to the difference analysis of *Lactobacillus* and *Gardnerella* in this study. So, we added a Welch's *t*-test on these two bacteria and found that the difference between the two groups was significant (*Lactobacillus, P*-value, 3.75 × 10^−5^; *Gardnerella, P*-value, 0.03). The results are displayed in Supplemental Data 3. Although it did not reach the threshold set of LEfSe analysis, because these species account for a large proportion and change greatly, it still has the significant role in the changes of vaginal microecology.

The recurrence rate of CA is as high as 59% and the time span for recurrence of CA varies from a few weeks to a few years. In this study, we also found *Mycoplasma, Chlamydia* and other microorganisms were higher in CA patients with HPV LR-6/11 infection. The accompanied infection of CA patients with other sexually transmitted diseases (AIDS, syphilis, gonorrhea, chlamydia and mycoplasma, genital herpes, etc.) is an important factors that led to the recurrence and a worse prognosis of CA (Mutoh et al., 2017; Klein et al., 2019). In addition, women with CA are often accompanied by urea mycoplasma (UU) infection (Binelli et al., 2012). In the treatment of CA patients, people often used a single sexually transmitted disease as the purpose of treatment, and ignored the mixed infection situation. This will not only cause a reduction cure rate of CA patients, but also a higher recurrence rate. Therefore, the detection of other sexually transmitted diseases in patients with CA is very important for prognosis, and the prevention of recurrence of CA is difficult to carry out. The high recurrence of CA is an urgent problem to be solved in clinical treatment.

Taken together, our study found CA patients with LR-HPV infection are often accompanied by vaginal microecological imbalance and systematically demonstrated the diversity and community structure of vaginal microorganisms between CA patients with LR-HPV infection and healthy people. It may be that the microbiota of LR subjects changed upon infection or upon development of CA or it may be that the LR subjects had a different microbiota and were more susceptible to infection or CA development. We hope this study will provide implications for the development of rational interventions against LR-HPV infection and CA, and will also provide a theoretical basis for vaginal microecological therapy.

## Data Availability

Publicly available datasets were analyzed in this study. This data can be found here: https://www.ncbi.nlm.nih.gov/sra/PRJNA548879.

## Ethics Statement

This study was undertaken with the approval of the Jinan Central Hospital Affiliated to Shandong University Ethics Service Committee. All experiments were carried out in accordance with the approved guidelines. Informed consent was obtained from all the participants prior to sampling.

## Author Contributions

YZ and YW conceived and directed the study. LW, FP, and MJ analyzed the data. FZ and YS made the clinical diagnoses. QZ, YH, XW, and JT collected and extracted the DNA. YZ was the major contributor in writing the manuscript. All authors read and approved the final manuscript.

### Conflict of Interest Statement

The author YZ is currently a postdoctoral fellow jointly trained by Shandong University and Shandong LaiBo Biotechnology Co., Ltd. The remaining authors declare that the research was conducted in the absence of any commercial or financial relationships that could be construed as a potential conflict of interest.

## Supplementary Material

Supplemental Figure 1 Analysis of the differences in the vaginal microbiome between LR HPV-6 and HPV-11. **(A)** Difference between and within groups was assessed by one-way analysis of similarities (ANOSIM) analysis. **(B,C)** Shannon diversity and Simpson diversity representing community richness were calculated. **(D,E)** The difference in the abundance of vaginal microbial communities at the phylum and genus level. **(F)** Spearman correlation coefficient analysis of dominant species. **(G)** Comparisons of gut bacteria between the LR6 and LR11 groups. The histogram shows the LDA score computed for genera differentially abundant between groups and identified using LEfSe.

Supplemental Data 2 Results of LEfSe's analysis (containing *P*-value for all species).

Supplemental Data 3 Welch's *t*-test for *Lactobacillus* and *Gardnerella*.
