# Supplementary figures and images for: Patients With LR-HPV Infection Have a Distinct Vaginal Microbiota in Comparison With Healthy Controls
Source: Front Cell Infect Microbiol. 2019 Aug 28;9:294. doi: 10.3389/fcimb.2019.00294 (PMC6722871; doi:10.3389/fcimb.2019.00294)

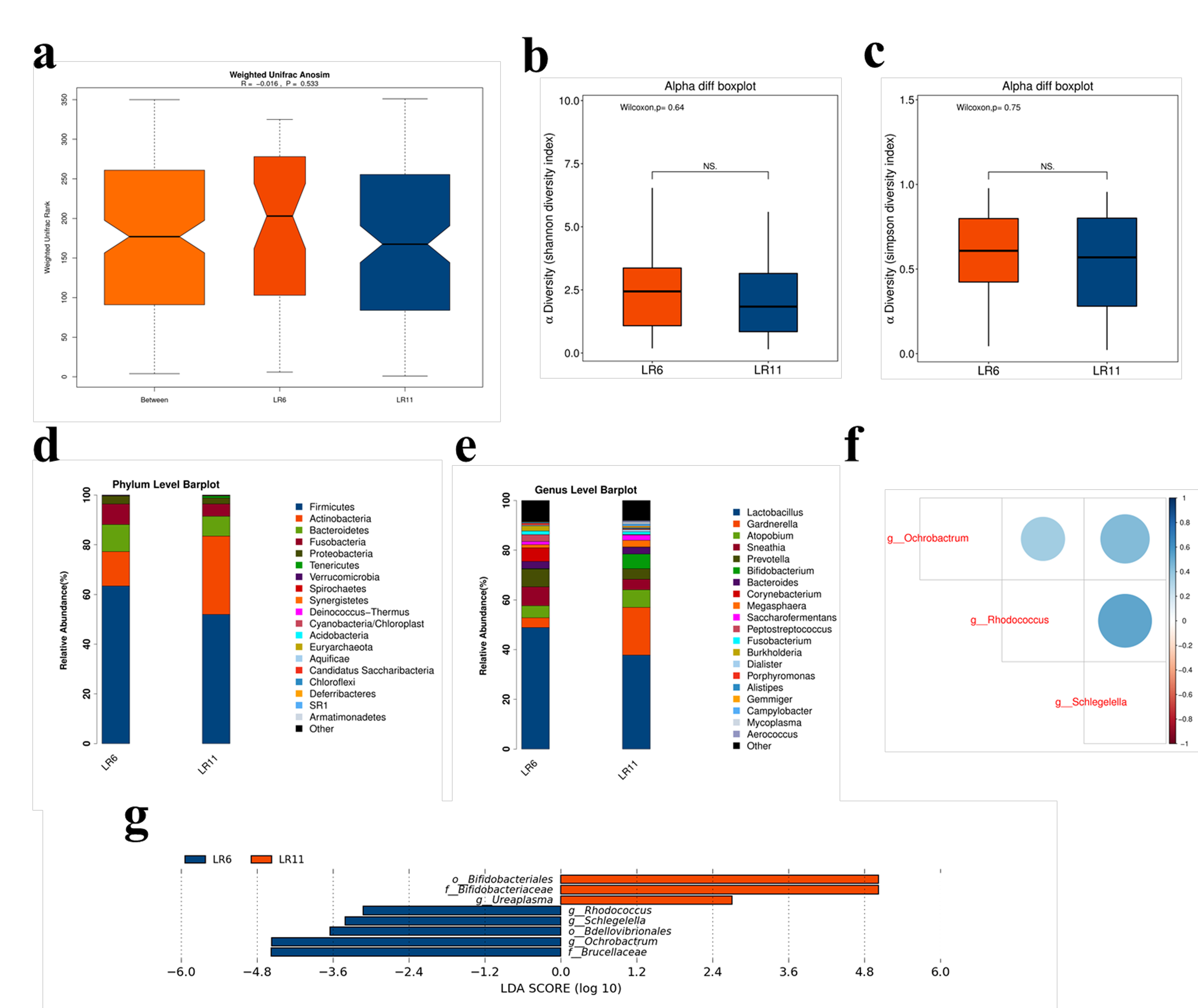

Supplement: Supplemental Figure 1 — Analysis of the differences in the vaginal microbiome between LR HPV-6 and HPV-11. (A) Difference between and within groups was assessed by one-way analysis of similarities (ANOSIM) analysis. (B,C) Shannon diversity and Simpson diversity representing community richness were calculated. (D,E) The difference in the abundance of vaginal microbial communities at the phylum and genus level. (F) Spearman correlation coefficient analysis of dominant species. (G) Comparisons of gut bacteria between the LR6 and LR11 groups. The histogram shows the LDA score computed for genera differentially abundant between groups and identified using LEfSe. [file Image_1.tif]

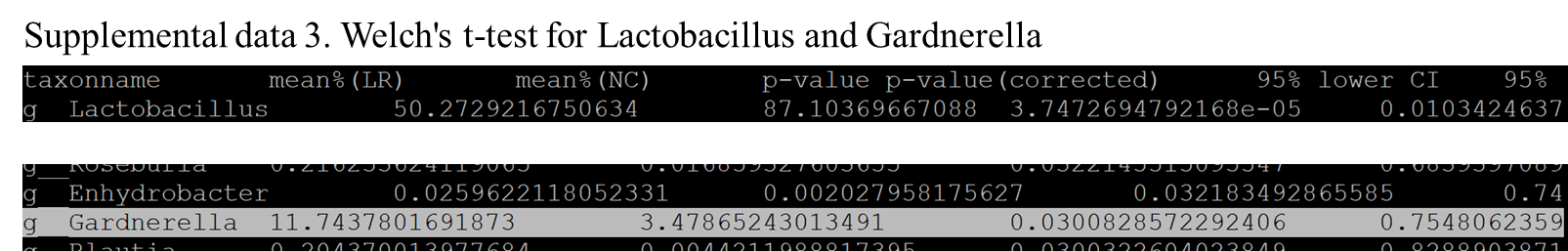

Supplement: Supplemental Data 3 — Welch's t-test for Lactobacillus and Gardnerella. [file Image_3.png]
