# Supplementary material for: Patients With LR-HPV Infection Have a Distinct Vaginal Microbiota in Comparison With Healthy Controls
Source: Front Cell Infect Microbiol. 2019 Aug 28;9:294. doi: 10.3389/fcimb.2019.00294 (PMC6722871; doi:10.3389/fcimb.2019.00294)
Supplement: Supplemental Data 2 — Results of LEfSe's analysis (containing P-value for all species). [file Image_2.pdf]

Supplemental data 2: Results of LEfSe's analysis (containing P value for all species).

1	species		P value
2	k__Bacteria.p__Fusobacteria.c__Fusobacteriia.o__Fusobacteriales.f__Leptotrichiaceae.g__Leptotrichia	LR	0.49015296
3	k__Bacteria.p__Proteobacteria.c__Alphaproteobacteria.o__Rhizobiales	LR	0.882806112
4	k__Archaea.p__Euryarchaeota.c__Methanomicrobia.o__Methanomicrobiales.f__Methanomicrobiaceae	LR	0.605484266
5	k__Bacteria.p__Verrucomicrobia.c__Opitutae.o__Puniceicoccales	LR	0.49015296
6	k__Bacteria.p__Proteobacteria.c__Betaproteobacteria.o__Neisseriales.f__Neisseriaceae.g__Neisseria	LR	0.230399242
7	k__Bacteria.p__Proteobacteria.c__Betaproteobacteria.o__Burkholderiales.f__Oxalobacteraceae.g__Massilia	NC	0.869215916
8	k__Bacteria.p__Proteobacteria.c__Alphaproteobacteria.o__Sphingomonadales.f__Erythrobacteraceae	LR	0.837153416
9	k__Bacteria.p__Firmicutes.c__Bacilli.o__Lactobacillales.f__Aerococcaceae.g__Aerococcus	LR	0.764714821
10	k__Archaea.p__Euryarchaeota.c__Methanomicrobia.o__Methanosarcinales	NC	0.701965334
11	k__Bacteria.p__Cyanobacteria_Chloroplast	LR	0.210305063
12	k__Bacteria.p__Firmicutes.c__Clostridia.o__Clostridiales.f__Clostridiales_Incertae Sedis XI.g__Tepidimicrobium	LR	0.325181041
13	k__Bacteria.p__Firmicutes.c__Erysipelotrichia.o__Erysipelotrichales.f__Erysipelotrichaceae.g__Erysipelotrichaceae_incertae_sedis	LR	0.110834514
14	k__Bacteria.p__Bacteroidetes.c__Bacteroidia.o__Bacteroidales.f__Porphyromonadaceae.g__Parabacteroides	LR	0.233535256
15	k__Bacteria.p__SR1.g__SR1_genera_incertae_sedis	LR	0.49015296
16	k__Bacteria.p__Proteobacteria.c__Betaproteobacteria.o__Burkholderiales.f__Sutterellaceae.g__Parasutterella	LR	0.210217675
17	k__Bacteria.p__Bacteroidetes.c__Bacteroidia.o__Bacteroidales	LR	0.065083346
18	k__Bacteria.p__Proteobacteria.c__Gammaproteobacteria.o__Xanthomonadales.f__Xanthomonadaceae.g__Stenotrophomonas	LR	0.536603334
19	k__Bacteria.p__Actinobacteria.c__Actinobacteria.o__Actinomycetales.f__Nocardiaceae.g__Rhodococcus	LR	0.931446714
20	k__Bacteria.p__Proteobacteria.c__Betaproteobacteria.o__Burkholderiales.f__Sutterellaceae.g__Sutterella	LR	0.0147318
21	k__Bacteria.p__Fusobacteria.c__Fusobacteriia.o__Fusobacteriales.f__Leptotrichiaceae.g__Sneathia	LR	0.004874163
22	k__Bacteria.p__Firmicutes.c__Bacilli.o__Lactobacillales.f__Streptococcaceae	LR	0.511876788
23	k__Bacteria.p__Firmicutes.c__Bacilli.o__Bacillales.f__Paenibacillaceae 1	NC	0.209344115
24	k__Bacteria.p__Firmicutes.c__Negativicutes.o__Selenomonadales.f__Veillonellaceae.g__Dialister	LR	0.019246472
25	k__Archaea.p__Thaumarchaeota	NC	0.038817136
26	k__Bacteria.p__Proteobacteria.c__Deltaproteobacteria.o__Desulfovibrionales	LR	0.737791916
27	k__Bacteria.p__Firmicutes.c__Bacilli.o__Lactobacillales.f__Lactobacillaceae.g__Lactobacillus	NC	0.068443717
28	k__Bacteria.p__Proteobacteria.c__Alphaproteobacteria.o__Rhizobiales.f__Methylobacteriaceae.g__Methylobacterium	LR	0.289218322
29	k__Bacteria.p__Acidobacteria.c__Acidobacteria_Gp4	LR	0.523616008
30	k__Bacteria.p__Acidobacteria.c__Acidobacteria_Gp5	NC	0.147299139
31	k__Bacteria.p__Acidobacteria.c__Acidobacteria_Gp6	NC	0.01828523
32	k__Bacteria.p__Acidobacteria.c__Acidobacteria_Gp7	LR	0.49015296
33	k__Bacteria.p__Acidobacteria.c__Acidobacteria_Gp1	LR	0.325181041
34	k__Bacteria.p__Proteobacteria.c__Deltaproteobacteria.o__Desulfovibrionales.f__Desulfovibrionaceae.g__Desulfovibrio	LR	0.133000928
35	k__Bacteria.p__Acidobacteria.c__Acidobacteria_Gp3	NC	0.038817136
36	k__Bacteria.p__Firmicutes.c__Bacilli.o__Bacillales.f__Bacillales_Incertae Sedis X	NC	0.147299139
37	k__Bacteria.p__Aquificae.c__Aquificae.o__Aquificales.f__Aquificaceae	LR	0.605484266
38	k__Bacteria.p__Deinococcus_Thermus.c__Deinococci.o__Thermales.f__Thermaceae.g__Thermus	LR	0.523616008
39	k__Bacteria.p__Firmicutes.c__Clostridia.o__Clostridiales.f__Ruminococcaceae.g__Fastidiosipila	NC	0.147299139
40	k__Bacteria.p__Bacteroidetes.c__Bacteroidia.o__Bacteroidales.f__Prevotellaceae.g__Paraprevotella	LR	0.688366193
41	k__Bacteria.p__Actinobacteria.c__Actinobacteria.o__Actinomycetales.f__Intrasporangiaceae.g__Janibacter	LR	0.78405657
42	k__Bacteria.p__Actinobacteria.c__Actinobacteria.o__Actinomycetales.f__Micrococcaceae	LR	0.495536748
43	k__Bacteria.p__Firmicutes.c__Bacilli.o__Lactobacillales.f__Carnobacteriaceae.g__Atopostipes	LR	0.49015296
44	k__Bacteria.p__Tenericutes.c__Mollicutes.o__Anaeroplasmatales	LR	0.49015296
45	k__Bacteria.p__Firmicutes.c__Clostridia.o__Clostridiales.f__Lachnospiraceae.g__Dorea	LR	0.108422889
46	k__Bacteria.p__Actinobacteria.c__Actinobacteria.o__Actinomycetales.f__Intrasporangiaceae.g__Arsenicicoccus	LR	0.49015296
47	k__Bacteria.p__Firmicutes.c__Bacilli.o__Lactobacillales.f__Lactobacillaceae	NC	0.068443717
48	k__Archaea.p__Euryarchaeota.c__Methanomicrobia.o__Methanosarcinales.f__Methanotrichaceae.g__Methanothrix	LR	0.325181041
49	k__Bacteria.p__Firmicutes.c__Negativicutes.o__Selenomonadales.f__Veillonellaceae.g__Anaerovibrio	NC	0.147299139
50	k__Bacteria.p__Proteobacteria	LR	0.187626774
51	k__Bacteria.p__Actinobacteria.c__Actinobacteria.o__Coriobacteriales.f__Coriobacteriaceae.g__Enterorhabdus	LR	0.49015296
52	k__Bacteria.p__Actinobacteria.c__Actinobacteria.o__Gaiellales.f__Gaiellaceae	NC	0.605484266
53	k__Bacteria.p__Cyanobacteria_Chloroplast.c__Chloroplast.f__Chloroplast.g__Streptophyta	LR	0.144275736
54	k__Bacteria.p__Firmicutes.c__Bacilli.o__Bacillales.f__Planococcaceae	LR	0.157247068
55	k__Bacteria.p__Actinobacteria.c__Actinobacteria.o__Actinomycetales.f__Intrasporangiaceae.g__Ornithinimicrobium	NC	0.935448572
56	k__Bacteria.p__Proteobacteria.c__Gammaproteobacteria.o__Legionellales.f__Coxiellaceae.g__Diplorickettsia	NC	0.147299139
57	k__Bacteria.p__Verrucomicrobia.c__Subdivision3	LR	0.325181041
58	k__Bacteria.p__Proteobacteria.c__Gammaproteobacteria.o__Pasteurellales	LR	0.00137041
59	k__Archaea.p__Euryarchaeota.c__Methanobacteria.o__Methanobacteriales	LR	0.110834514
60	k__Bacteria.p__Acidobacteria.c__Acidobacteria_Gp4.g__Blastocatella	LR	0.49015296
61	k__Bacteria.p__Actinobacteria.c__Actinobacteria.o__Actinomycetales.f__Mycobacteriaceae.g__Mycobacterium	LR	0.157247068
62	k__Bacteria.p__Firmicutes.c__Clostridia.o__Clostridiales.f__Clostridiales_Incertae Sedis XI.g__Finegoldia	LR	0.9550667
63	k__Bacteria.p__Bacteroidetes.c__Flavobacteriia	LR	0.265123342
64	k__Bacteria.p__Aquificae.c__Aquificae	LR	0.605484266
65	k__Bacteria.p__Proteobacteria.c__Betaproteobacteria.o__Burkholderiales.f__Alcaligenaceae.g__Achromobacter	LR	0.49015296
66	k__Bacteria.p__Firmicutes.c__Clostridia.o__Clostridiales.f__Ruminococcaceae.g__Ruminococcus	LR	0.04662429
67	k__Bacteria.p__Tenericutes.c__Mollicutes	LR	0.048461978
68	k__Bacteria.p__Proteobacteria.c__Gammaproteobacteria.o__Legionellales.f__Legionellaceae	LR	0.325181041
69	k__Bacteria.p__Proteobacteria.c__Alphaproteobacteria.o__Rhizobiales.f__Brucellaceae	LR	0.220107639
70	k__Bacteria.p__Bacteroidetes.c__Sphingobacteriia	LR	0.763751461
71	k__Bacteria.p__Deferribacteres.c__Deferribacteres.o__Deferribacterales	LR	0.49015296
72	k__Bacteria.p__Proteobacteria.c__Gammaproteobacteria.o__Pasteurellales.f__Pasteurellaceae.g__Aggregatibacter		-
73	k__Bacteria.p__Bacteroidetes.c__Flavobacteriia.o__Flavobacteriales.f__Flavobacteriaceae.g__Cloacibacterium	LR	0.289465675
74	k__Bacteria.p__Firmicutes.c__Clostridia.o__Clostridiales.f__Peptostreptococcaceae.g__Peptostreptococcaceae_incertae_sedis	LR	0.49015296
75	k__Bacteria.p__Proteobacteria.c__Deltaproteobacteria	LR	0.695895895
76	k__Bacteria.p__Firmicutes.c__Clostridia.o__Clostridiales.f__Lachnospiraceae.g__Anaerostipes	LR	0.050264034
77	k__Bacteria.p__Proteobacteria.c__Betaproteobacteria.o__Burkholderiales.f__Comamonadaceae.g__Schlegelella	LR	0.137014843
78	k__Bacteria.p__Firmicutes.c__Negativicutes.o__Selenomonadales	LR	0.117209786
79	k__Bacteria.p__Proteobacteria.c__Alphaproteobacteria.o__Sphingomonadales	LR	0.197877103
80	k__Bacteria.p__Bacteroidetes.c__Flavobacteriia.o__Flavobacteriales	LR	0.265123342
81	k__Bacteria.p__Actinobacteria.c__Actinobacteria.o__Actinomycetales.f__Pseudonocardiaceae.g__Saccharopolyspora		-
82	k__Bacteria.p__Actinobacteria.c__Actinobacteria.o__Actinomycetales.f__Actinomycetaceae.g__Arcanobacterium	LR	0.110834514
83	k__Bacteria.p__Acidobacteria.c__Acidobacteria_Gp4.g__Gp4	LR	0.967697825
84	k__Bacteria.p__Actinobacteria.c__Actinobacteria.o__Actinomycetales.f__Micromonosporaceae	NC	0.147299139
85	k__Bacteria.p__Proteobacteria.c__Gammaproteobacteria.o__Enterobacteriales.f__Enterobacteriaceae.g__Enterobacter	LR	0.900818035
86	k__Bacteria.p__Bacteroidetes.c__Flavobacteriia.o__Flavobacteriales.f__Flavobacteriaceae.g__Epilithonimonas	LR	0.325181041

87	k__Bacteria.p__Deinococcus_Thermus.c__Deinococci.o__Deinococcales.f__Deinococcaceae	LR	0.310304766
88	k__Bacteria.p__Actinobacteria.c__Actinobacteria.o__Coriobacteriales.f__Coriobacteriaceae.g__Slackia	LR	0.157247068
89	k__Bacteria.p__Proteobacteria.c__Gammaproteobacteria.o__Pseudomonadales.f__Moraxellaceae.g__Alkanindiges	NC	0.147299139
90	k__Bacteria.p__Actinobacteria.c__Actinobacteria.o__Actinomycetales.f__Demequinaceae	NC	0.147299139
91	k__Bacteria.p__Actinobacteria.c__Actinobacteria.o__Actinomycetales.f__Streptomycetaceae.g__Streptomyces	LR	0.49015296
92	k__Bacteria.p__Proteobacteria.c__Alphaproteobacteria.o__Sphingomonadales.f__Sphingomonadaceae.g__Sphingobium	LR	0.49015296
93	k__Bacteria.p__Proteobacteria.c__Alphaproteobacteria.o__Rhodospirillales.f__Acetobacteraceae.g__Roseomonas	LR	0.967697825
94	k__Bacteria.p__Firmicutes.c__Bacilli.o__Lactobacillales.f__Carnobacteriaceae.g__Carnobacterium	NC	0.147299139
95	k__Bacteria.p__Proteobacteria.c__Betaproteobacteria.o__Neisseriales.f__Neisseriaceae	LR	0.539429695
96	k__Bacteria.p__Proteobacteria.c__Betaproteobacteria.o__Burkholderiales.f__Burkholderiaceae.g__Cupriavidus	LR	0.078204718
97	k__Bacteria.p__Proteobacteria.c__Alphaproteobacteria.o__Rhizobiales.f__Hyphomicrobiaceae.g__Devosia	LR	0.605484266
98	k__Bacteria.p__Bacteroidetes	LR	0.073132571
99	k__Bacteria.p__Verrucomicrobia.c__Verrucomicrobiae.o__Verrucomicrobiales	NC	0.425148371
100	k__Bacteria.p__Proteobacteria.c__Gammaproteobacteria.o__Enterobacteriales.f__Enterobacteriaceae.g__Providencia	NC	0.195021369
101	k__Bacteria.p__Actinobacteria.c__Actinobacteria.o__Coriobacteriales.f__Coriobacteriaceae.g__Collinsella	LR	0.43504359
102	k__Bacteria.p__Bacteroidetes.c__Bacteroidia.o__Bacteroidales.f__Porphyromonadaceae.g__Paludibacter	LR	0.49015296
103	k__Bacteria.p__Firmicutes.c__Clostridia.o__Clostridiales.f__Peptostreptococcaceae.g__Filifactor	LR	0.49015296
104	k__Bacteria.p__Actinobacteria.c__Actinobacteria.o__Actinomycetales.f__Streptomycetaceae	LR	0.49015296
105	k__Bacteria.p__Gemmatimonadetes.c__Gemmatimonadetes.o__Gemmatimonadales	NC	0.209344115
106	k__Bacteria.p__Actinobacteria.c__Actinobacteria.o__Actinomycetales.f__Microbacteriaceae	LR	0.634869245
107	k__Bacteria.p__Proteobacteria.c__Betaproteobacteria.o__Burkholderiales.f__Comamonadaceae.g__Acidovorax	LR	0.49015296
108	k__Bacteria.p__Actinobacteria.c__Actinobacteria.o__Acidimicrobiales	LR	0.605484266
109	k__Bacteria.p__Proteobacteria.c__Gammaproteobacteria.o__Pasteurellales.f__Pasteurellaceae	LR	0.00137041
110	k__Bacteria.p__Actinobacteria.c__Actinobacteria.o__Rubrobacterales.f__Rubrobacteraceae	LR	0.325181041
111	k__Bacteria.p__Firmicutes.c__Clostridia.o__Clostridiales.f__Syntrophomonadaceae	LR	0.49015296
112	k__Bacteria.p__Fusobacteria.c__Fusobacteriia.o__Fusobacteriales.f__Fusobacteriaceae.g__Cetobacterium	LR	0.49015296
113	k__Archaea.p__Euryarchaeota.c__Thermoplasmata	LR	0.49015296
114	k__Archaea.p__Euryarchaeota	LR	0.477915856
115	k__Bacteria.p__Acidobacteria.c__Acidobacteria_Gp16	LR	0.49015296
116	k__Bacteria.p__Firmicutes.c__Erysipelotrichia.o__Erysipelotrichales.f__Erysipelotrichaceae.g__Clostridium XVIII	LR	0.018698637
117	k__Bacteria.p__Actinobacteria.c__Actinobacteria.o__Actinomycetales.f__Pseudonocardiaceae.g__Pseudonocardia	LR	0.49015296
118	k__Bacteria.p__Acidobacteria.c__Acidobacteria_Gp10	NC	0.147299139
119	k__Bacteria.p__Proteobacteria.c__Alphaproteobacteria.o__Rhizobiales.f__Bradyrhizobiaceae.g__Bradyrhizobium	LR	0.49015296
120	k__Bacteria.p__Bacteroidetes.c__Flavobacteriia.o__Flavobacteriales.f__Flavobacteriaceae.g__Chryseobacterium	LR	0.102157492
121	k__Bacteria.p__Proteobacteria.c__Epsilonproteobacteria.o__Campylobacterales.f__Helicobacteraceae	NC	0.010726951
122	k__Bacteria.p__Proteobacteria.c__Deltaproteobacteria.o__Bdellovibrionales.f__Bacteriovoracaceae	LR	0.49015296
123	k__Archaea.p__Euryarchaeota.c__Methanomicrobia.o__Methanosarcinales.f__Methanosarcinaceae	NC	0.436619026
124	k__Bacteria.p__Firmicutes.c__Erysipelotrichia	LR	0.005466975
125	k__Bacteria.p__Aminicenantes.g__Aminicenantes_genera_incertae_sedis	LR	0.49015296
126	k__Bacteria.p__Proteobacteria.c__Betaproteobacteria.o__Burkholderiales.f__Burkholderiaceae	LR	0.0466895
127	k__Bacteria.p__Proteobacteria.c__Gammaproteobacteria.o__Xanthomonadales.f__Xanthomonadaceae.g__Lysobacter	NC	0.038817136
128	k__Bacteria.p__Firmicutes.c__Bacilli.o__Bacillales.f__Bacillales_Incertae Sedis XI.g__Gemella	LR	0.462572059
129	k__Bacteria.p__Proteobacteria.c__Gammaproteobacteria.o__Pseudomonadales	NC	0.009794117
130	k__Bacteria.p__Proteobacteria.c__Betaproteobacteria.o__Burkholderiales.f__Alcaligenaceae.g__Alcaligenes	LR	0.967697825
131	k__Archaea.p__Euryarchaeota.c__Methanomicrobia	LR	0.583641776
132	k__Bacteria.p__Firmicutes.c__Bacilli.o__Lactobacillales.f__Carnobacteriaceae.g__Granulicatella	LR	0.21949913
133	k__Bacteria.p__Firmicutes.c__Negativicutes.o__Selenomonadales.f__Veillonellaceae.g__Megamonas	LR	0.235438853
134	k__Bacteria.p__Bacteroidetes.c__Bacteroidia.o__Bacteroidales.f__Bacteroidaceae	LR	0.182181226
135	k__Bacteria.p__Bacteroidetes.c__Cytophagia	LR	0.49015296
136	k__Bacteria.p__Verrucomicrobia.c__Opitutae.o__Opitutales.f__Opitutaceae	LR	0.49015296
137	k__Bacteria.p__Actinobacteria.c__Actinobacteria.o__Actinomycetales.f__Actinomycetaceae.g__Mobiluncus	LR	0.897092061
138	k__Bacteria.p__Verrucomicrobia.c__Verrucomicrobiae.o__Verrucomicrobiales.f__Verrucomicrobiaceae	NC	0.425148371
139	k__Bacteria.p__Proteobacteria.c__Gammaproteobacteria.o__Pseudomonadales.f__Moraxellaceae.g__Acinetobacter	LR	0.776550328
140	k__Bacteria.p__Tenericutes.c__Mollicutes.o__Mycoplasmatales.f__Mycoplasmataceae.g__Ureaplasma	LR	0.167495739
141	k__Bacteria.p__Gemmatimonadetes	NC	0.209344115
142	k__Bacteria.p__Actinobacteria.c__Actinobacteria.o__Actinomycetales.f__Micrococcaceae.g__Enteractinococcus	LR	0.49015296
143	k__Bacteria.p__Proteobacteria.c__Alphaproteobacteria.o__Sphingomonadales.f__Sphingomonadaceae.g__Novosphingobium	LR	0.555725072
144	k__Bacteria.p__Proteobacteria.c__Betaproteobacteria.o__Burkholderiales.f__Comamonadaceae.g__Delftia	LR	0.016628137
145	k__Bacteria.p__Proteobacteria.c__Gammaproteobacteria.o__Aeromonadales.f__Succinivibrionaceae.g__Succinivibrio	NC	0.010734105
146	k__Bacteria.p__Firmicutes.c__Clostridia.o__Clostridiales.f__Clostridiales_Incertae Sedis XI.g__Parvimonas	LR	0.053087246
147	k__Bacteria.p__Deferribacteres.c__Deferribacteres.o__Deferribacterales.f__Deferribacteraceae.g__Mucispirillum	LR	0.49015296
148	k__Bacteria.p__Proteobacteria.c__Deltaproteobacteria.o__Desulfobacterales.f__Desulfobulbaceae.g__Desulfobulbus	LR	0.49015296
149	k__Bacteria.p__Proteobacteria.c__Alphaproteobacteria.o__Rhodospirillales.f__Rhodospirillaceae	NC	0.935448572
150	k__Bacteria.p__Actinobacteria.c__Actinobacteria.o__Actinomycetales.f__Cellulomonadaceae	NC	0.147299139
151	k__Bacteria.p__Proteobacteria.c__Gammaproteobacteria.o__Enterobacteriales.f__Enterobacteriaceae.g__Proteus	LR	0.224413712
152	k__Bacteria.p__Bacteroidetes.c__Bacteroidia	LR	0.065083346
153	k__Bacteria.p__Firmicutes.c__Bacilli.o__Lactobacillales.f__Aerococcaceae.g__Facklamia	LR	0.49015296
154	k__Bacteria.p__Proteobacteria.c__Betaproteobacteria.o__Burkholderiales.f__Alcaligenaceae	LR	0.967697825
155	k__Bacteria.p__Firmicutes.c__Bacilli.o__Lactobacillales.f__Leuconostocaceae	NC	0.147299139
156	k__Bacteria.p__Actinobacteria.c__Actinobacteria.o__Rubrobacterales	LR	0.325181041
157	k__Bacteria.p__Firmicutes.c__Bacilli.o__Bacillales.f__Staphylococcaceae.g__Staphylococcus	LR	0.574622011
158	k__Bacteria.p__Firmicutes.c__Bacilli.o__Bacillales.f__Bacillaceae 1	LR	0.55339351
159	k__Bacteria.p__Aquificae	LR	0.605484266
160	k__Bacteria.p__Proteobacteria.c__Alphaproteobacteria.o__Rhodobacterales.f__Rhodobacteraceae.g__Paracoccus	NC	0.371555029
161	k__Bacteria.p__Actinobacteria	LR	0.117325188
162	k__Bacteria.p__Actinobacteria.c__Actinobacteria.o__Coriobacteriales.f__Coriobacteriaceae.g__Atopobium	LR	0.963343696
163	k__Bacteria.p__Cloacimonetes	LR	0.49015296
164	k__Bacteria.p__Firmicutes.c__Negativicutes.o__Selenomonadales.f__Acidaminococcaceae	LR	0.057958795
165	k__Archaea.p__Euryarchaeota.c__Methanomicrobia.o__Methanosarcinales.f__Methanosarcinaceae.g__Methanosarcina	NC	0.436619026
166	k__Bacteria.p__Bacteroidetes.c__Sphingobacteriia.o__Sphingobacteriales.f__Chitinophagaceae		-
167	k__Bacteria.p__Acidobacteria.c__Acidobacteria_Gp5.g__Gp5	NC	0.147299139
168	k__Bacteria.p__Acidobacteria.c__Acidobacteria_Gp17	NC	0.605484266
169	k__Archaea.p__Euryarchaeota.c__Methanobacteria.o__Methanobacteriales.f__Methanobacteriaceae	LR	0.110834514
170	k__Bacteria.p__Bacteroidetes.c__Bacteroidia.o__Bacteroidales.f__Porphyromonadaceae.g__Odoribacter	LR	0.323336949
171	k__Bacteria.p__Proteobacteria.c__Alphaproteobacteria.o__Rhodobacterales.f__Rhodobacteraceae	NC	0.270298588

172	None	LR	0.158731782
173	k__Bacteria.p__Proteobacteria.c__Betaproteobacteria.o__Burkholderiales.f__Comamonadaceae.g__Comamonas	LR	0.362824418
174	k__Bacteria.p__Actinobacteria.c__Actinobacteria.o__Actinomycetales.f__Actinomycetaceae.g__Varibaculum	LR	0.49015296
175	k__Bacteria.p__Firmicutes.c__Clostridia.o__Clostridiales.f__Lachnospiraceae.g__Fusicatenibacter	LR	0.008785811
176	k__Archaea.p__Euryarchaeota.c__Methanomicrobia.o__Methanomicrobiales.f__Methanoregulaceae	LR	0.49015296
177	k__Bacteria.p__Bacteroidetes.c__Bacteroidia.o__Bacteroidales.f__Porphyromonadaceae.g__Porphyromonas	LR	0.897578358
178	k__Bacteria.p__Bacteroidetes.c__Sphingobacteriia.o__Sphingobacteriales.f__Rhodothermaceae	LR	0.49015296
179	k__Bacteria.p__Tenericutes.c__Mollicutes.o__Anaeroplasmatales.f__Anaeroplasmataceae.g__Anaeroplasma	LR	0.49015296
180	k__Bacteria.p__Firmicutes.c__Negativicutes.o__Selenomonadales.f__Veillonellaceae	LR	0.156703828
181	k__Bacteria.p__Firmicutes	NC	0.103882827
182	k__Bacteria.p__Spirochaetes.c__Spirochaetia.o__Spirochaetales.f__Spirochaetaceae.g__Treponema	LR	0.325181041
183	k__Bacteria.p__Actinobacteria.c__Actinobacteria.o__Actinomycetales.f__Propionibacteriaceae.g__Propionibacterium	LR	0.871174224
184	k__Bacteria	NC	0.734199581
185	k__Bacteria.p__Cyanobacteria_Chloroplast.c__Cyanobacteria		-
186	k__Bacteria.p__Deferribacteres.c__Deferribacteres	LR	0.49015296
187	k__Bacteria.p__Firmicutes.c__Clostridia.o__Clostridiales.f__Incertae Sedis XI	NC	0.104729509
188	k__Bacteria.p__Proteobacteria.c__Alphaproteobacteria.o__Rhizobiales.f__Bradyrhizobiaceae.g__Bosea	LR	0.325181041
189	k__Bacteria.p__Firmicutes.c__Negativicutes	LR	0.117209786
190	k__Bacteria.p__Actinobacteria.c__Actinobacteria.o__Actinomycetales.f__Micrococcaceae.g__Arthrobacter	NC	0.209344115
191	k__Bacteria.p__Proteobacteria.c__Alphaproteobacteria.o__Rhizobiales.f__Rhodobiaceae.g__Parvibaculum	LR	0.49015296
192	k__Bacteria.p__Firmicutes.c__Clostridia.o__Clostridiales.f__Incertae Sedis XI.g__Murdochiella	NC	0.104729509
193	k__Bacteria.p__Spirochaetes.c__Spirochaetia.o__Spirochaetales	LR	0.325181041
194	k__Bacteria.p__Aquificae.c__Aquificae.o__Aquificales	LR	0.605484266
195	k__Bacteria.p__Bacteroidetes.c__Cytophagia.o__Cytophagales.f__Cytophagaceae.g__Adhaeribacter	LR	0.49015296
196	k__Bacteria.p__Verrucomicrobia.c__Opitutae	LR	0.325181041
197	k__Bacteria.p__Firmicutes.c__Clostridia.o__Clostridiales.f__Lachnospiraceae.g__Clostridium XlVa	LR	0.223480451
198	k__Bacteria.p__Firmicutes.c__Clostridia.o__Clostridiales.f__Lachnospiraceae.g__Clostridium XlVb	LR	0.173463436
199	k__Bacteria.p__Firmicutes.c__Bacilli.o__Bacillales.f__Bacillaceae 1.g__Anoxybacillus	LR	0.736932768
200	k__Bacteria.p__Proteobacteria.c__Epsilonproteobacteria.o__Campylobacterales	LR	0.486861099
201	k__Bacteria.p__Tenericutes.c__Mollicutes.o__Anaeroplasmatales.f__Anaeroplasmataceae	LR	0.49015296
202	k__Bacteria.p__Proteobacteria.c__Gammaproteobacteria.o__Legionellales.f__Legionellaceae.g__Legionella	LR	0.325181041
203	k__Bacteria.p__Proteobacteria.c__Alphaproteobacteria.o__Rhizobiales.f__Hyphomicrobiaceae.g__Pedomicrobium	NC	0.147299139
204	k__Bacteria.p__Proteobacteria.c__Betaproteobacteria.o__Burkholderiales.f__Burkholderiaceae.g__Ralstonia	LR	0.110834514
205	k__Bacteria.p__Bacteroidetes.c__Bacteroidia.o__Bacteroidales.f__Porphyromonadaceae.g__Butyricimonas	LR	0.02776022
206	k__Bacteria.p__Firmicutes.c__Clostridia.o__Clostridiales.f__Ruminococcaceae.g__Flavonifractor	LR	0.078204718
207	k__Bacteria.p__Firmicutes.c__Clostridia.o__Clostridiales.f__Clostridiales_Incertae Sedis XI	LR	0.24400668
208	k__Bacteria.p__Firmicutes.c__Clostridia.o__Clostridiales.f__Lachnospiraceae.g__Blautia	LR	0.01002
209	k__Bacteria.p__Proteobacteria.c__Gammaproteobacteria.o__Cardiobacteriales	NC	0.571581844
210	k__Bacteria.p__Actinobacteria.c__Actinobacteria.o__Actinomycetales.f__Propionibacteriaceae.g__Propionimicrobium	NC	0.147299139
211	k__Bacteria.p__Proteobacteria.c__Betaproteobacteria.o__Burkholderiales.f__Comamonadaceae.g__Pelomonas	LR	0.983844131
212	k__Bacteria.p__Firmicutes.c__Clostridia.o__Clostridiales.f__Ruminococcaceae.g__Ethanoligenens	LR	0.49015296
213	k__Bacteria.p__Verrucomicrobia.c__Verrucomicrobiae.o__Verrucomicrobiales.f__Verrucomicrobiaceae.g__Akkermansia	NC	0.435065618
214	k__Bacteria.p__Proteobacteria.c__Alphaproteobacteria.o__Caulobacterales.f__Caulobacteraceae.g__Phenylobacterium	NC	0.479444928
215	k__Bacteria.p__Firmicutes.c__Clostridia.o__Clostridiales.f__Peptostreptococcaceae.g__Clostridium XI	LR	0.013769314
216	k__Bacteria.p__Proteobacteria.c__Betaproteobacteria.o__Burkholderiales.f__Oxalobacteraceae.g__Herbaspirillum	LR	0.224413712
217	k__Bacteria.p__Firmicutes.c__Bacilli.o__Bacillales.f__Bacillaceae 1.g__Geobacillus	LR	0.078204718
218	k__Bacteria.p__Firmicutes.c__Negativicutes.o__Selenomonadales.f__Acidaminococcaceae.g__Acidaminococcus	LR	0.49015296
219	k__Bacteria.p__Synergistetes.c__Synergistia	LR	0.162237376
220	k__Bacteria.p__Proteobacteria.c__Alphaproteobacteria.o__Rhizobiales.f__Hyphomicrobiaceae	LR	0.209344115
221	k__Bacteria.p__Firmicutes.c__Bacilli.o__Lactobacillales.f__Carnobacteriaceae.g__Dolosigranulum	LR	0.49015296
222	k__Bacteria.p__Actinobacteria.c__Actinobacteria.o__Actinomycetales.f__Demequinaceae.g__Demequina	NC	0.147299139
223	k__Bacteria.p__Firmicutes.c__Bacilli.o__Bacillales.f__Planococcaceae.g__Chryseomicrobium	LR	0.224413712
224	k__Bacteria.p__Acidobacteria.c__Acidobacteria_Gp17.g__Gp17	NC	0.605484266
225	k__Bacteria.p__Verrucomicrobia.c__Opitutae.o__Opitutales.f__Opitutaceae.g__Alterococcus	LR	0.49015296
226	k__Bacteria.p__Aminicenantes	LR	0.49015296
227	k__Archaea.p__Thaumarchaeota.o__Nitrososphaerales	NC	0.038817136
228	k__Archaea.p__Euryarchaeota.c__Methanobacteria.o__Methanobacteriales.f__Methanobacteriaceae.g__Methanobrevibacter	LR	0.110834514
229	k__Bacteria.p__Acidobacteria	LR	0.572511585
230	k__Bacteria.p__Firmicutes.c__Bacilli.o__Bacillales.f__Paenibacillaceae 1.g__Paenibacillus	LR	0.605484266
231	k__Bacteria.p__Cloacimonetes.g__Candidatus Cloacamonas	LR	0.49015296
232	k__Bacteria.p__Actinobacteria.c__Actinobacteria.o__Actinomycetales.f__Nocardioidaceae.g__Nocardioides	LR	0.325116021
233	k__Bacteria.p__Actinobacteria.c__Actinobacteria.o__Gaiellales	NC	0.605484266
234	k__Bacteria.p__Firmicutes.c__Bacilli.o__Lactobacillales.f__Leuconostocaceae.g__Weissella	NC	0.147299139
235	k__Bacteria.p__Proteobacteria.c__Alphaproteobacteria.o__Rhizobiales.f__Rhizobiaceae	LR	0.879779377
236	k__Bacteria.p__Synergistetes	LR	0.162237376
237	k__Bacteria.p__Firmicutes.c__Clostridia.o__Clostridiales.f__Lachnospiraceae.g__Lachnospiracea_incertae_sedis	LR	0.031198449
238	k__Bacteria.p__Acidobacteria.c__Acidobacteria_Gp3.g__Gp3	NC	0.038817136
239	k__Bacteria.p__Firmicutes.c__Clostridia.o__Clostridiales.f__Eubacteriaceae.g__Acetobacterium	LR	0.49015296
240	k__Bacteria.p__Synergistetes.c__Synergistia.o__Synergistales.f__Synergistaceae.g__Pyramidobacter	LR	0.325181041
241	k__Bacteria.p__Proteobacteria.c__Epsilonproteobacteria.o__Campylobacterales.f__Campylobacteraceae	LR	0.992975791
242	k__Bacteria.p__Actinobacteria.c__Actinobacteria.o__Actinomycetales.f__Corynebacteriaceae.g__Corynebacterium	LR	0.152669991
243	k__Bacteria.p__Firmicutes.c__Clostridia.o__Clostridiales.f__Peptococcaceae 1.g__Peptococcus	LR	0.503067899
244	k__Bacteria.p__Firmicutes.c__Clostridia.o__Clostridiales.f__Clostridiales_Incertae Sedis XIII	LR	0.49015296
245	k__Bacteria.p__Proteobacteria.c__Deltaproteobacteria.o__Desulfobacterales	LR	0.49015296
246	k__Bacteria.p__Bacteroidetes.c__Flavobacteriia.o__Flavobacteriales.f__Flavobacteriaceae.g__Flavobacterium	NC	0.038817136
247	k__Bacteria.p__Cyanobacteria_Chloroplast.c__Cyanobacteria.f__Family VIII.g__GpVIII	LR	0.49015296
248	k__Bacteria.p__Tenericutes.c__Mollicutes.o__Mycoplasmatales.f__Mycoplasmataceae.g__Mycoplasma	LR	0.068162674
249	k__Bacteria.p__Firmicutes.c__Bacilli.o__Bacillales.f__Staphylococcaceae.g__Jeotgalicoccus		-
250	k__Bacteria.p__Verrucomicrobia.c__Spartobacteria	LR	0.605484266
251	k__Bacteria.p__Bacteroidetes.c__Bacteroidia.o__Bacteroidales.f__Prevotellaceae.g__Alloprevotella	LR	0.32157415
252	k__Bacteria.p__Actinobacteria.c__Actinobacteria.o__Actinomycetales.f__Micrococcaceae.g__Kocuria	LR	0.191753215
253	k__Bacteria.p__Actinobacteria.c__Actinobacteria.o__Actinomycetales.f__Actinomycetaceae	LR	0.046643504
254	k__Bacteria.p__Actinobacteria.c__Actinobacteria.o__Rubrobacterales.f__Rubrobacteraceae.g__Rubrobacter	LR	0.325181041
255	k__Archaea.p__Euryarchaeota.c__Methanomicrobia.o__Methanomicrobiales.f__Methanomicrobiaceae.g__Methanoculleus	LR	0.605484266
256	k__Bacteria.p__Deinococcus_Thermus.c__Deinococci.o__Thermales	LR	0.523616008
257	k__Bacteria.p__Firmicutes.c__Clostridia.o__Clostridiales.f__Lachnospiraceae	LR	0.00338422

258	k__Bacteria.p__Firmicutes.c__Bacilli.o__Bacillales.f__Staphylococcaceae	LR	0.692785741
259	k__Bacteria.p__Actinobacteria.c__Actinobacteria.o__Actinomycetales.f__Microbacteriaceae.g__Microbacterium	LR	0.614186409
260	k__Bacteria.p__Bacteroidetes.c__Bacteroidia.o__Bacteroidales.f__Rikenellaceae.g__Alistipes	LR	0.042528059
261	k__Bacteria.p__Bacteroidetes.c__Sphingobacteriia.o__Sphingobacteriales.f__Rhodothermaceae.g__Rhodothermus	LR	0.49015296
262	k__Bacteria.p__Bacteroidetes.c__Bacteroidia.o__Bacteroidales.f__Porphyromonadaceae.g__Dysgonomonas	LR	0.49015296
263	k__Bacteria.p__Firmicutes.c__Clostridia.o__Clostridiales.f__Lachnospiraceae.g__Oribacterium	LR	0.49015296
264	k__Bacteria.p__Firmicutes.c__Clostridia.o__Clostridiales.f__Lachnospiraceae.g__Ruminococcus2	LR	0.018025403
265	k__Bacteria.p__Firmicutes.c__Clostridia.o__Clostridiales.f__Clostridiaceae 1	LR	0.16357066
266	k__Bacteria.p__Bacteroidetes.c__Flavobacteriia.o__Flavobacteriales.f__Flavobacteriaceae	LR	0.265123342
267	k__Bacteria.p__Fusobacteria.c__Fusobacteriia.o__Fusobacteriales.f__Fusobacteriaceae.g__Fusobacterium	LR	0.125366456
268	k__Bacteria.p__Proteobacteria.c__Betaproteobacteria.o__Hydrogenophilales	LR	0.000140433
269	k__Bacteria.p__Bacteroidetes.c__Bacteroidia.o__Bacteroidales.f__Porphyromonadaceae.g__Coprobacter	LR	0.00398306
270	k__Bacteria.p__Firmicutes.c__Clostridia.o__Clostridiales.f__Lachnospiraceae.g__Howardella	LR	0.169381279
271	k__Bacteria.p__Proteobacteria.c__Gammaproteobacteria.o__Oceanospirillales.f__Halomonadaceae.g__Halomonas	LR	0.325181041
272	k__Bacteria.p__Firmicutes.c__Clostridia.o__Clostridiales.f__Ruminococcaceae.g__Clostridium IV	LR	0.294184813
273	k__Bacteria.p__Deinococcus_Thermus.c__Deinococci.o__Deinococcales	LR	0.310304766
274	k__Bacteria.p__Fusobacteria.c__Fusobacteriia.o__Fusobacteriales.f__Leptotrichiaceae	LR	0.00272281
275	k__Bacteria.p__Proteobacteria.c__Alphaproteobacteria.o__Sphingomonadales.f__Sphingomonadaceae.g__Sphingopyxis	LR	0.224413712
276	k__Bacteria.p__Proteobacteria.c__Alphaproteobacteria.o__Rhodospirillales	NC	0.869399023
277	k__Archaea	LR	0.797107599
278	k__Bacteria.p__Actinobacteria.c__Actinobacteria.o__Gaiellales.f__Gaiellaceae.g__Gaiella	NC	0.605484266
279	k__Bacteria.p__Proteobacteria.c__Betaproteobacteria.o__Burkholderiales.f__Burkholderiales_incertae_sedis.g__Tepidimonas	NC	0.052683721
280	k__Bacteria.p__Actinobacteria.c__Actinobacteria.o__Acidimicrobiales.f__Acidimicrobineae_incertae_sedis	LR	0.49015296
281	k__Bacteria.p__Actinobacteria.c__Actinobacteria.o__Actinomycetales.f__Dermabacteraceae	LR	0.110834514
282	k__Archaea.p__Thaumarchaeota.o__Nitrososphaerales.f__Nitrososphaeraceae.g__Nitrososphaera	NC	0.038817136
283	k__Bacteria.p__Actinobacteria.c__Actinobacteria.o__Actinomycetales.f__Pseudonocardiaceae	LR	0.967697825
284	k__Bacteria.p__Firmicutes.c__Clostridia.o__Clostridiales.f__Syntrophomonadaceae.g__Syntrophomonas	LR	0.49015296
285	k__Bacteria.p__Proteobacteria.c__Gammaproteobacteria.o__Oceanospirillales	LR	0.325181041
286	k__Bacteria.p__Firmicutes.c__Bacilli.o__Bacillales.f__Bacillales_Incertae Sedis XI	LR	0.462572059
287	k__Bacteria.p__Proteobacteria.c__Betaproteobacteria.o__Burkholderiales.f__Oxalobacteraceae.g__Undibacterium	NC	0.147299139
288	k__Bacteria.p__Firmicutes.c__Clostridia.o__Clostridiales.f__Ruminococcaceae.g__Faecalibacterium	LR	0.275574337
289	k__Bacteria.p__Actinobacteria.c__Actinobacteria.o__Coriobacteriales	LR	0.184918313
290	k__Bacteria.p__Proteobacteria.c__Alphaproteobacteria.o__Rhizobiales.f__Brucellaceae.g__Ochrobactrum	LR	0.220107639
291	k__Bacteria.p__Firmicutes.c__Clostridia.o__Clostridiales.f__Peptococcaceae 1	LR	0.503067899
292	k__Bacteria.p__Nitrospirae.c__Nitrospira	NC	0.147299139
293	k__Bacteria.p__Firmicutes.c__Bacilli.o__Bacillales.f__Staphylococcaceae.g__Salinicoccus	LR	0.49015296
294	k__Bacteria.p__Proteobacteria.c__Alphaproteobacteria.o__Sphingomonadales.f__Sphingomonadaceae.g__Sphingomonas	NC	0.187385274
295	k__Archaea.p__Euryarchaeota.c__Methanomicrobia.o__Methanomicrobiales.f__Methanospirillaceae.g__Methanospirillum	LR	0.49015296
296	k__Bacteria.p__Proteobacteria.c__Alphaproteobacteria.o__Rhizobiales.f__Bradyrhizobiaceae	LR	0.96487883
297	k__Bacteria.p__Firmicutes.c__Clostridia.o__Clostridiales.f__Ruminococcaceae.g__Gemmiger	LR	0.024646945
298	k__Bacteria.p__Synergistetes.c__Synergistia.o__Synergistales	LR	0.162237376
299	k__Bacteria.p__Proteobacteria.c__Gammaproteobacteria.o__Enterobacteriales.f__Enterobacteriaceae.g__Klebsiella	LR	0.198589056
300	k__Bacteria.p__Spirochaetes	LR	0.325181041
301	k__Bacteria.p__Fusobacteria	LR	0.001150683
302	k__Archaea.p__Euryarchaeota.c__Methanomicrobia.o__Methanomicrobiales		-
303	k__Bacteria.p__Firmicutes.c__Negativicutes.o__Selenomonadales.f__Veillonellaceae.g__Negativicoccus	LR	0.191034252
304	k__Bacteria.p__Candidatus Saccharibacteria	NC	0.065653783
305	k__Bacteria.p__Firmicutes.c__Bacilli.o__Lactobacillales.f__Aerococcaceae.g__Eremococcus	LR	0.325181041
306	k__Bacteria.p__Firmicutes.c__Clostridia.o__Clostridiales.f__Clostridiaceae 1.g__Caloramator	LR	0.325181041
307	k__Bacteria.p__Nitrospirae.c__Nitrospira.o__Nitrospirales.f__Nitrospiraceae.g__Nitrospira	NC	0.147299139
308	k__Bacteria.p__Actinobacteria.c__Actinobacteria.o__Actinomycetales.f__Cellulomonadaceae.g__Cellulomonas	NC	0.147299139
309	k__Bacteria.p__Firmicutes.c__Clostridia.o__Clostridiales.f__Lachnospiraceae.g__Moryella	LR	0.49015296
310	k__Bacteria.p__Proteobacteria.c__Gammaproteobacteria.o__Enterobacteriales.f__Enterobacteriaceae.g__Cronobacter	LR	0.49015296
311	k__Bacteria.p__Actinobacteria.c__Actinobacteria.o__Actinomycetales.f__Nocardiaceae	LR	0.931446714
312	k__Bacteria.p__Proteobacteria.c__Alphaproteobacteria.o__Caulobacterales.f__Caulobacteraceae.g__Brevundimonas	LR	0.266701601
313	k__Bacteria.p__Proteobacteria.c__Betaproteobacteria.o__Burkholderiales.f__Burkholderiales_incertae_sedis	NC	0.052683721
314	k__Bacteria.p__Actinobacteria.c__Actinobacteria.o__Actinomycetales.f__Brevibacteriaceae.g__Brevibacterium		-
315	k__Bacteria.p__Verrucomicrobia.c__Opitutae.o__Opitutales	LR	0.49015296
316	k__Bacteria.p__Actinobacteria.c__Actinobacteria.o__Bifidobacteriales.f__Bifidobacteriaceae	LR	0.261899794
317	k__Bacteria.p__Proteobacteria.c__Betaproteobacteria.o__Burkholderiales.f__Sutterellaceae	LR	0.04731605
318	k__Bacteria.p__Proteobacteria.c__Betaproteobacteria.o__Hydrogenophilales.f__Hydrogenophilaceae	LR	0.000140433
319	k__Bacteria.p__Firmicutes.c__Clostridia.o__Clostridiales.f__Lachnospiraceae.g__Coprococcus	LR	0.003337708
320	k__Bacteria.p__Proteobacteria.c__Betaproteobacteria.o__Methylophilales.f__Methylophilaceae	LR	0.49015296
321	k__Bacteria.p__Firmicutes.c__Clostridia.o__Clostridiales.f__Clostridiaceae 1.g__Clostridium sensu stricto	LR	0.280963556
322	k__Bacteria.p__Actinobacteria.c__Actinobacteria.o__Bifidobacteriales	LR	0.261899794
323	k__Bacteria.p__Spirochaetes.c__Spirochaetia	LR	0.325181041
324	k__Bacteria.p__Nitrospirae.c__Nitrospira.o__Nitrospirales.f__Nitrospiraceae	NC	0.147299139
325	k__Bacteria.p__Firmicutes.c__Negativicutes.o__Selenomonadales.f__Veillonellaceae.g__Veillonella	LR	0.277131728
326	k__Bacteria.p__Firmicutes.c__Clostridia.o__Clostridiales.f__Ruminococcaceae.g__Hydrogenoanaerobacterium	LR	0.49015296
327	k__Bacteria.p__Bacteroidetes.c__Bacteroidia.o__Bacteroidales.f__Prevotellaceae.g__Prevotella	LR	0.06179454
328	k__Bacteria.p__Gemmatimonadetes.c__Gemmatimonadetes.o__Gemmatimonadales.f__Gemmatimonadaceae.g__Gemmatimonas	NC	0.209344115
329	k__Bacteria.p__Firmicutes.c__Clostridia.o__Clostridiales.f__Clostridiales_Incertae Sedis XI.g__Peptoniphilus	LR	0.251026271
330	k__Bacteria.p__Proteobacteria.c__Alphaproteobacteria.o__Rhizobiales.f__Methylobacteriaceae	LR	0.289218322
331	k__Bacteria.p__Proteobacteria.c__Alphaproteobacteria.o__Rhizobiales.f__Rhizobiaceae.g__Rhizobium	LR	0.879779377
332	k__Bacteria.p__Proteobacteria.c__Gammaproteobacteria.o__Pasteurellales.f__Pasteurellaceae.g__Pasteurella	LR	0.000285982
333	k__Bacteria.p__Firmicutes.c__Clostridia.o__Clostridiales.f__Clostridiales_Incertae Sedis XI.g__Gallicola	LR	0.49015296
334	k__Bacteria.p__Firmicutes.c__Clostridia.o__Clostridiales.f__Lachnospiraceae.g__Lachnoanaerobaculum	LR	0.49015296
335	k__Bacteria.p__Proteobacteria.c__Betaproteobacteria.o__Burkholderiales.f__Comamonadaceae	LR	0.010265103
336	k__Bacteria.p__Firmicutes.c__Bacilli.o__Lactobacillales.f__Aerococcaceae.g__Abiotrophia	LR	0.224413712
337	k__Bacteria.p__Proteobacteria.c__Alphaproteobacteria.o__Caulobacterales.f__Caulobacteraceae	LR	0.844786137
338	k__Bacteria.p__Firmicutes.c__Negativicutes.o__Selenomonadales.f__Veillonellaceae.g__Allisonella	LR	0.49015296
339	k__Bacteria.p__Tenericutes	LR	0.048461978
340	k__Archaea.p__Euryarchaeota.c__Methanomicrobia.o__Methanomicrobiales.f__Methanospirillaceae	LR	0.49015296
341	k__Bacteria.p__Proteobacteria.c__Betaproteobacteria.o__Burkholderiales.f__Oxalobacteraceae	NC	0.898921217
342	k__Bacteria.p__Actinobacteria.c__Actinobacteria.o__Actinomycetales.f__Propionibacteriaceae	LR	0.955060886
343	k__Bacteria.p__Proteobacteria.c__Betaproteobacteria.o__Burkholderiales.f__Burkholderiaceae.g__Burkholderia	LR	0.565943678
344	k__Bacteria.p__Firmicutes.c__Clostridia.o__Clostridiales.f__Eubacteriaceae.g__Eubacterium		-
345	k__Bacteria.p__Firmicutes.c__Erysipelotrichia.o__Erysipelotrichales.f__Erysipelotrichaceae.g__Solobacterium	LR	0.49015296

346	k__Bacteria.p__Proteobacteria.c__Betaproteobacteria.o__Neisseriales	LR	0.539429695
347	k__Bacteria.p__Deferribacteres.c__Deferribacteres.o__Deferribacterales.f__Deferribacteraceae	LR	0.49015296
348	k__Bacteria.p__Firmicutes.c__Bacilli.o__Lactobacillales.f__Enterococcaceae	NC	0.26633711
349	k__Bacteria.p__Firmicutes.c__Bacilli.o__Bacillales.f__Bacillales_Incertae Sedis X.g__Thermicanus	NC	0.147299139
350	k__Bacteria.p__Firmicutes.c__Clostridia.o__Clostridiales.f__Eubacteriaceae		-
351	k__Bacteria.p__Deinococcus_Thermus.c__Deinococci	LR	0.34170034
352	k__Bacteria.p__Acidobacteria.c__Acidobacteria_Gp6.g__Gp6	NC	0.01828523
353	k__Bacteria.p__Bacteroidetes.c__Cytophagia.o__Cytophagales.f__Cytophagaceae	LR	0.49015296
354	k__Bacteria.p__Proteobacteria.c__Gammaproteobacteria.o__Xanthomonadales.f__Xanthomonadaceae	LR	0.546584932
355	k__Bacteria.p__Proteobacteria.c__Deltaproteobacteria.o__Bdellovibrionales.f__Bacteriovoracaceae.g__Peredibacter	LR	0.49015296
356	k__Bacteria.p__Firmicutes.c__Clostridia.o__Clostridiales.f__Clostridiaceae 1.g__Proteiniclasticum	LR	0.49015296
357	k__Bacteria.p__Verrucomicrobia.c__Spartobacteria.g__Spartobacteria_genera_incertae_sedis	LR	0.605484266
358	k__Bacteria.p__Candidatus Saccharibacteria.g__Saccharibacteria_genera_incertae_sedis	NC	0.065653783
359	k__Bacteria.p__Aquificae.c__Aquificae.o__Aquificales.f__Aquificaceae.g__Hydrogenobacter	LR	0.605484266
360	k__Bacteria.p__Actinobacteria.c__Actinobacteria.o__Bifidobacteriales.f__Bifidobacteriaceae.g__Bifidobacterium	LR	0.025159478
361	k__Bacteria.p__Actinobacteria.c__Actinobacteria.o__Actinomycetales.f__Micrococcaceae.g__Rothia	LR	0.736932768
362	k__Bacteria.p__Bacteroidetes.c__Bacteroidia.o__Bacteroidales.f__Bacteroidaceae.g__Bacteroides	LR	0.182181226
363	k__Bacteria.p__Proteobacteria.c__Gammaproteobacteria.o__Enterobacteriales.f__Enterobacteriaceae	LR	0.739789554
364	k__Bacteria.p__Firmicutes.c__Clostridia.o__Clostridiales.f__Peptostreptococcaceae	LR	0.001278036
365	k__Bacteria.p__Firmicutes.c__Clostridia.o__Clostridiales.f__Clostridiales_Incertae Sedis XI.g__Anaerococcus	LR	0.375653399
366	k__Bacteria.p__Cyanobacteria_Chloroplast.c__Chloroplast	LR	0.144275736
367	k__Bacteria.p__Firmicutes.c__Negativicutes.o__Selenomonadales.f__Veillonellaceae.g__Selenomonas	LR	0.325181041
368	k__Bacteria.p__Firmicutes.c__Negativicutes.o__Selenomonadales.f__Veillonellaceae.g__Sporolituus	LR	0.49015296
369	k__Bacteria.p__Actinobacteria.c__Actinobacteria	LR	0.117325188
370	k__Bacteria.p__Proteobacteria.c__Alphaproteobacteria	LR	0.441403371
371	k__Bacteria.p__Synergistetes.c__Synergistia.o__Synergistales.f__Synergistaceae	LR	0.162237376
372	k__Bacteria.p__Firmicutes.c__Erysipelotrichia.o__Erysipelotrichales.f__Erysipelotrichaceae.g__Holdemania	LR	0.49015296
373	k__Bacteria.p__Actinobacteria.c__Actinobacteria.o__Actinomycetales.f__Corynebacteriaceae	LR	0.152669991
374	k__Bacteria.p__Firmicutes.c__Bacilli.o__Bacillales.f__Bacillaceae 1.g__Bacillus	LR	0.654834065
375	k__Archaea.p__Thaumarchaeota.o__Nitrososphaerales.f__Nitrososphaeraceae	NC	0.038817136
376	k__Bacteria.p__Firmicutes.c__Erysipelotrichia.o__Erysipelotrichales.f__Erysipelotrichaceae.g__Bulleidia	LR	0.503067899
377	k__Bacteria.p__Firmicutes.c__Erysipelotrichia.o__Erysipelotrichales.f__Erysipelotrichaceae.g__Turicibacter	LR	0.078204718
378	k__Bacteria.p__Bacteroidetes.c__Sphingobacteriia.o__Sphingobacteriales.f__Sphingobacteriaceae.g__Solitalea	NC	0.147299139
379	k__Bacteria.p__Firmicutes.c__Clostridia.o__Clostridiales.f__Clostridiales_Incertae Sedis XIII.g__Anaerovorax	LR	0.49015296
380	k__Bacteria.p__Proteobacteria.c__Gammaproteobacteria.o__Pasteurellales.f__Pasteurellaceae.g__Haemophilus	LR	0.144275736
381	k__Bacteria.p__Acidobacteria.c__Acidobacteria_Gp7.g__Gp7	LR	0.49015296
382	k__Bacteria.p__Proteobacteria.c__Gammaproteobacteria.o__Aeromonadales.f__Succinivibrionaceae	NC	0.010734105
383	k__Bacteria.p__Proteobacteria.c__Gammaproteobacteria.o__Cardiobacteriales.f__Cardiobacteriaceae.g__Suttonella	NC	0.147299139
384	k__Bacteria.p__Firmicutes.c__Clostridia.o__Clostridiales	LR	0.004639362
385	k__Bacteria.p__Firmicutes.c__Bacilli.o__Bacillales.f__Planococcaceae.g__Sporosarcina	LR	0.49015296
386	k__Bacteria.p__Actinobacteria.c__Actinobacteria.o__Actinomycetales.f__Intrasporangiaceae	LR	0.717496599
387	k__Bacteria.p__Proteobacteria.c__Deltaproteobacteria.o__Syntrophobacterales.f__Syntrophaceae	LR	0.49015296
388	k__Bacteria.p__Proteobacteria.c__Deltaproteobacteria.o__Myxococcales.f__Polyangiaceae	LR	0.49015296
389	k__Bacteria.p__Bacteroidetes.c__Bacteroidia.o__Bacteroidales.f__Porphyromonadaceae.g__Barnesiella	LR	0.171173889
390	k__Bacteria.p__Proteobacteria.c__Gammaproteobacteria.o__Xanthomonadales	LR	0.546584932
391	k__Bacteria.p__Actinobacteria.c__Actinobacteria.o__Acidimicrobiales.f__Acidimicrobineae_incertae_sedis.g__Aciditerrimonas	LR	0.49015296
392	k__Bacteria.p__Proteobacteria.c__Deltaproteobacteria.o__Bdellovibrionales.f__Bdellovibrionaceae	LR	0.224413712
393	k__Archaea.p__Euryarchaeota.c__Thermoplasmata.o__Methanomassiliicoccales.f__Methanomassiliicoccaceae.g__Methanomassiliicoccus	LR	0.49015296
394	k__Bacteria.p__Actinobacteria.c__Actinobacteria.o__Actinomycetales.f__Dermabacteraceae.g__Brachybacterium	LR	0.224413712
395	k__Bacteria.p__Proteobacteria.c__Deltaproteobacteria.o__Syntrophobacterales	LR	0.49015296
396	k__Bacteria.p__Firmicutes.c__Erysipelotrichia.o__Erysipelotrichales.f__Erysipelotrichaceae	LR	0.005466975
397	k__Bacteria.p__Firmicutes.c__Clostridia.o__Clostridiales.f__Ruminococcaceae	LR	0.000622956
398	k__Bacteria.p__Synergistetes.c__Synergistia.o__Synergistales.f__Synergistaceae.g__Lactivibrio	LR	0.49015296
399	k__Bacteria.p__Actinobacteria.c__Actinobacteria.o__Actinomycetales.f__Actinomycetaceae.g__Actinomyces	LR	0.017523676
400	k__Bacteria.p__Fusobacteria.c__Fusobacteriia	LR	0.001150683
401	k__Bacteria.p__Firmicutes.c__Negativicutes.o__Selenomonadales.f__Acidaminococcaceae.g__Phascolarctobacterium	LR	0.036350835
402	k__Bacteria.p__Proteobacteria.c__Deltaproteobacteria.o__Myxococcales.f__Kofleriaceae	LR	0.49015296
403	k__Bacteria.p__Firmicutes.c__Clostridia.o__Clostridiales.f__Ruminococcaceae.g__Oscillibacter	LR	0.057220596
404	k__Archaea.p__Euryarchaeota.c__Thermoplasmata.o__Methanomassiliicoccales.f__Methanomassiliicoccaceae	LR	0.49015296
405	k__Bacteria.p__Proteobacteria.c__Gammaproteobacteria.o__Pseudomonadales.f__Moraxellaceae.g__Moraxella	LR	0.49015296
406	k__Bacteria.p__Proteobacteria.c__Alphaproteobacteria.o__Alphaproteobacteria_incertae_sedis.g__Geminicoccus	NC	0.147299139
407	k__Bacteria.p__Nitrospirae	NC	0.147299139
408	k__Bacteria.p__Actinobacteria.c__Actinobacteria.o__Actinomycetales.f__Nocardioidaceae	LR	0.325116021
409	k__Bacteria.p__Firmicutes.c__Clostridia.o__Clostridiales.f__Ruminococcaceae.g__Anaerotruncus		-
410	k__Bacteria.p__Proteobacteria.c__Deltaproteobacteria.o__Myxococcales	LR	0.325181041
411	k__Bacteria.p__Firmicutes.c__Clostridia.o__Clostridiales.f__Lachnospiraceae.g__Catonella	LR	0.325181041
412	k__Bacteria.p__Proteobacteria.c__Deltaproteobacteria.o__Desulfobacterales.f__Desulfobulbaceae	LR	0.49015296
413	k__Bacteria.p__Proteobacteria.c__Deltaproteobacteria.o__Desulfovibrionales.f__Desulfovibrionaceae	LR	0.724878013
414	k__Bacteria.p__Firmicutes.c__Erysipelotrichia.o__Erysipelotrichales	LR	0.005466975
415	k__Bacteria.p__Proteobacteria.c__Deltaproteobacteria.o__Syntrophobacterales.f__Syntrophaceae.g__Smithella	LR	0.49015296
416	k__Bacteria.p__Proteobacteria.c__Gammaproteobacteria.o__Pseudomonadales.f__Moraxellaceae.g__Enhydrobacter	LR	0.012031579
417	k__Bacteria.p__Armatimonadetes.g__Armatimonadetes_gp7	LR	0.49015296
418	k__Bacteria.p__Bacteroidetes.c__Bacteroidia.o__Bacteroidales.f__Prevotellaceae	LR	0.07427752
419	k__Bacteria.p__Bacteroidetes.c__Flavobacteriia.o__Flavobacteriales.f__Flavobacteriaceae.g__Capnocytophaga	LR	0.71043071
420	k__Bacteria.p__Synergistetes.c__Synergistia.o__Synergistales.f__Synergistaceae.g__Jonquetella	LR	0.068602496
421	k__Archaea.p__Euryarchaeota.c__Thermoplasmata.o__Methanomassiliicoccales	LR	0.49015296
422	k__Bacteria.p__Firmicutes.c__Clostridia.o__Clostridiales.f__Peptostreptococcaceae.g__Peptostreptococcus	LR	0.02006036
423	k__Bacteria.p__Verrucomicrobia	NC	0.287255912
424	k__Bacteria.p__Cyanobacteria_Chloroplast.c__Chloroplast.f__Chloroplast	LR	0.144275736
425	k__Bacteria.p__Proteobacteria.c__Alphaproteobacteria.o__Rhodobacterales.f__Rhodobacteraceae.g__Rubellimicrobium	LR	0.713809239
426	k__Bacteria.p__Proteobacteria.c__Epsilonproteobacteria.o__Campylobacterales.f__Helicobacteraceae.g__Helicobacter	NC	0.010726951
427	k__Bacteria.p__Proteobacteria.c__Alphaproteobacteria.o__Alphaproteobacteria_incertae_sedis	NC	0.147299139
428	k__Bacteria.p__Proteobacteria.c__Gammaproteobacteria.o__Pseudomonadales.f__Moraxellaceae.g__Psychrobacter	LR	0.544586271
429	k__Archaea.p__Euryarchaeota.c__Methanomicrobia.o__Methanosarcinales.f__Methanotrichaceae	LR	0.325181041
430	k__Bacteria.p__Proteobacteria.c__Alphaproteobacteria.o__Sphingomonadales.f__Sphingomonadaceae	LR	0.147779058
431	k__Bacteria.p__Proteobacteria.c__Deltaproteobacteria.o__Syntrophobacterales.f__Syntrophaceae.g__Syntrophus	LR	0.49015296

432	k__Bacteria.p__Proteobacteria.c__Epsilonproteobacteria.o__Campylobacterales.f__Campylobacteraceae.g__Arcobacter	LR	0.325181041
433	k__Bacteria.p__Proteobacteria.c__Betaproteobacteria.o__Burkholderiales	LR	0.101137485
434	k__Bacteria.p__Bacteroidetes.c__Sphingobacteriia.o__Sphingobacteriales.f__Sphingobacteriaceae	NC	0.147299139
435	k__Bacteria.p__Proteobacteria.c__Betaproteobacteria.o__Burkholderiales.f__Comamonadaceae.g__Hydrogenophaga	LR	0.325181041
436	k__Bacteria.p__Firmicutes.c__Clostridia.o__Clostridiales.f__Lachnospiraceae.g__Roseburia	LR	0.040482247
437	k__Bacteria.p__Bacteroidetes.c__Bacteroidia.o__Bacteroidales.f__Rikenellaceae	LR	0.042528059
438	k__Bacteria.p__Firmicutes.c__Bacilli.o__Lactobacillales	NC	0.070759815
439	k__Bacteria.p__Verrucomicrobia.c__Subdivision3.g__Subdivision3_genera_incertae_sedis	LR	0.325181041
440	k__Bacteria.p__Proteobacteria.c__Betaproteobacteria.o__Methylophilales.f__Methylophilaceae.g__Methylobacillus	LR	0.49015296
441	k__Bacteria.p__Proteobacteria.c__Deltaproteobacteria.o__Bdellovibrionales	LR	0.157247068
442	k__Bacteria.p__SR1	LR	0.49015296
443	k__Archaea.p__Euryarchaeota.c__Methanobacteria	LR	0.110834514
444	k__Bacteria.p__Proteobacteria.c__Gammaproteobacteria.o__Enterobacteriales.f__Enterobacteriaceae.g__Pantoea	LR	0.669728864
445	k__Bacteria.p__Actinobacteria.c__Actinobacteria.o__Actinomycetales.f__Brevibacteriaceae		-
446	k__Bacteria.p__Proteobacteria.c__Betaproteobacteria	LR	0.051775885
447	k__Bacteria.p__Spirochaetes.c__Spirochaetia.o__Spirochaetales.f__Spirochaetaceae	LR	0.325181041
448	k__Bacteria.p__Firmicutes.c__Bacilli.o__Bacillales	LR	0.137535102
449	k__Bacteria.p__Cyanobacteria_Chloroplast.c__Cyanobacteria.f__Family VIII	LR	0.49015296
450	k__Bacteria.p__Bacteroidetes.c__Bacteroidia.o__Bacteroidales.f__Porphyromonadaceae	LR	0.398510428
451	k__Bacteria.p__Firmicutes.c__Clostridia	LR	0.004639362
452	k__Bacteria.p__Firmicutes.c__Clostridia.o__Clostridiales.f__Clostridiales_Incertae Sedis XI.g__Helcococcus	LR	0.49015296
453	k__Bacteria.p__Proteobacteria.c__Alphaproteobacteria.o__Rhizobiales.f__Rhodobiaceae	LR	0.49015296
454	k__Bacteria.p__Firmicutes.c__Negativicutes.o__Selenomonadales.f__Veillonellaceae.g__Schwartzia	LR	0.49015296
455	k__Bacteria.p__Fusobacteria.c__Fusobacteriia.o__Fusobacteriales	LR	0.001150683
456	k__Bacteria.p__Deferribacteres	LR	0.49015296
457	k__Bacteria.p__Fusobacteria.c__Fusobacteriia.o__Fusobacteriales.f__Fusobacteriaceae	LR	0.125366456
458	k__Bacteria.p__Tenericutes.c__Mollicutes.o__Mycoplasmatales.f__Mycoplasmataceae	LR	0.062171308
459	k__Bacteria.p__Verrucomicrobia.c__Opitutae.o__Puniceicoccales.f__Puniceicoccaceae	LR	0.49015296
460	k__Bacteria.p__Proteobacteria.c__Alphaproteobacteria.o__Rhizobiales.f__Bradyrhizobiaceae.g__Rhodopseudomonas	NC	0.188095311
461	k__Bacteria.p__Firmicutes.c__Bacilli	NC	0.070759815
462	k__Bacteria.p__Proteobacteria.c__Deltaproteobacteria.o__Bdellovibrionales.f__Bdellovibrionaceae.g__Vampirovibrio	LR	0.224413712
463	k__Bacteria.p__Firmicutes.c__Negativicutes.o__Selenomonadales.f__Veillonellaceae.g__Megasphaera	LR	0.016659455
464	k__Bacteria.p__Proteobacteria.c__Betaproteobacteria.o__Methylophilales	LR	0.49015296
465	k__Bacteria.p__Proteobacteria.c__Gammaproteobacteria.o__Pseudomonadales.f__Pseudomonadaceae.g__Pseudomonas	NC	0.007647174
466	k__Bacteria.p__Proteobacteria.c__Betaproteobacteria.o__Hydrogenophilales.f__Hydrogenophilaceae.g__Hydrogenophilus	LR	5.38E-05
467	k__Bacteria.p__Verrucomicrobia.c__Verrucomicrobiae.o__Verrucomicrobiales.f__Verrucomicrobiaceae.g__Luteolibacter	LR	0.49015296
468	k__Bacteria.p__Actinobacteria.c__Actinobacteria.o__Coriobacteriales.f__Coriobacteriaceae.g__Eggerthella	LR	0.110834514
469	k__Bacteria.p__Verrucomicrobia.c__Verrucomicrobiae	NC	0.425148371
470	k__Bacteria.p__Bacteroidetes.c__Sphingobacteriia.o__Sphingobacteriales	LR	0.763751461
471	k__Bacteria.p__Proteobacteria.c__Gammaproteobacteria.o__Legionellales	LR	0.983844131
472	k__Bacteria.p__Acidobacteria.c__Acidobacteria_Gp1.g__Gp1	LR	0.325181041
473	k__Bacteria.p__Synergistetes.c__Synergistia.o__Synergistales.f__Synergistaceae.g__Cloacibacillus	LR	0.49015296
474	k__Bacteria.p__Gemmatimonadetes.c__Gemmatimonadetes.o__Gemmatimonadales.f__Gemmatimonadaceae	NC	0.209344115
475	k__Bacteria.p__Proteobacteria.c__Gammaproteobacteria.o__Pseudomonadales.f__Moraxellaceae	LR	0.324444665
476	k__Bacteria.p__Firmicutes.c__Bacilli.o__Lactobacillales.f__Streptococcaceae.g__Streptococcus	LR	0.511876788
477	k__Bacteria.p__Proteobacteria.c__Deltaproteobacteria.o__Myxococcales.f__Kofleriaceae.g__Kofleria	LR	0.49015296
478	k__Bacteria.p__Firmicutes.c__Bacilli.o__Bacillales.f__Bacillaceae 1.g__Aeribacillus	LR	0.49015296
479	k__Bacteria.p__Acidobacteria.c__Acidobacteria_Gp16.g__Gp16	LR	0.49015296
480	k__Bacteria.p__Proteobacteria.c__Gammaproteobacteria.o__Aeromonadales	NC	0.010734105
481	k__Archaea.p__Euryarchaeota.c__Methanomicrobia.o__Methanomicrobiales.f__Methanoregulaceae.g__Methanosphaerula	LR	0.49015296
482	k__Bacteria.p__Firmicutes.c__Clostridia.o__Clostridiales.f__Ruminococcaceae.g__Butyricicoccus	LR	0.728162046
483	k__Bacteria.p__Proteobacteria.c__Epsilonproteobacteria.o__Campylobacterales.f__Campylobacteraceae.g__Campylobacter	LR	0.650193876
484	k__Bacteria.p__Proteobacteria.c__Gammaproteobacteria.o__Cardiobacteriales.f__Cardiobacteriaceae.g__Cardiobacterium	LR	0.49015296
485	k__Bacteria.p__Proteobacteria.c__Gammaproteobacteria.o__Enterobacteriales.f__Enterobacteriaceae.g__Morganella	LR	0.325181041
486	k__Bacteria.p__Armatimonadetes	LR	0.49015296
487	k__Bacteria.p__Deinococcus_Thermus	LR	0.34170034
488	k__Bacteria.p__Proteobacteria.c__Alphaproteobacteria.o__Rhodospirillales.f__Acetobacteraceae	LR	0.736932768
489	k__Bacteria.p__Proteobacteria.c__Gammaproteobacteria.o__Oceanospirillales.f__Halomonadaceae	LR	0.325181041
490	k__Bacteria.p__Nitrospirae.c__Nitrospira.o__Nitrospirales	NC	0.147299139
491	k__Bacteria.p__Deinococcus_Thermus.c__Deinococci.o__Deinococcales.f__Deinococcaceae.g__Deinococcus	LR	0.310304766
492	k__Bacteria.p__Proteobacteria.c__Betaproteobacteria.o__Burkholderiales.f__Comamonadaceae.g__Curvibacter	LR	0.605484266
493	k__Bacteria.p__Proteobacteria.c__Gammaproteobacteria	NC	0.022085937
494	k__Bacteria.p__Firmicutes.c__Bacilli.o__Lactobacillales.f__Carnobacteriaceae	LR	0.262995541
495	k__Bacteria.p__Bacteroidetes.c__Cytophagia.o__Cytophagales	LR	0.49015296
496	k__Bacteria.p__Acidobacteria.c__Acidobacteria_Gp10.g__Gp10	NC	0.147299139
497	k__Bacteria.p__Proteobacteria.c__Gammaproteobacteria.o__Pseudomonadales.f__Pseudomonadaceae	NC	0.007311074
498	k__Bacteria.p__Tenericutes.c__Mollicutes.o__Mycoplasmatales	LR	0.062171308
499	k__Bacteria.p__Gemmatimonadetes.c__Gemmatimonadetes	NC	0.209344115
500	k__Bacteria.p__Firmicutes.c__Clostridia.o__Clostridiales.f__Ruminococcaceae.g__Intestinimonas	LR	0.49015296
501	k__Bacteria.p__Proteobacteria.c__Deltaproteobacteria.o__Desulfovibrionales.f__Desulfovibrionaceae.g__Bilophila	LR	0.041166461
502	k__Bacteria.p__Actinobacteria.c__Actinobacteria.o__Coriobacteriales.f__Coriobacteriaceae	LR	0.184918313
503	k__Bacteria.p__Proteobacteria.c__Gammaproteobacteria.o__Enterobacteriales.f__Enterobacteriaceae.g__Escherichia_Shigella	LR	0.728722738
504	k__Bacteria.p__Actinobacteria.c__Actinobacteria.o__Bifidobacteriales.f__Bifidobacteriaceae.g__Gardnerella	LR	0.556891393
505	k__Bacteria.p__Proteobacteria.c__Alphaproteobacteria.o__Rhodospirillales.f__Rhodospirillaceae.g__Dongia	NC	0.935448572
506	k__Bacteria.p__Bacteroidetes.c__Bacteroidia.o__Bacteroidales.f__Porphyromonadaceae.g__Tannerella	LR	0.750287633
507	k__Bacteria.p__Proteobacteria.c__Alphaproteobacteria.o__Rhodobacterales	NC	0.270298588
508	k__Bacteria.p__Firmicutes.c__Bacilli.o__Lactobacillales.f__Enterococcaceae.g__Enterococcus	NC	0.26633711
509	k__Bacteria.p__Proteobacteria.c__Gammaproteobacteria.o__Enterobacteriales	LR	0.739789554
510	k__Bacteria.p__Proteobacteria.c__Alphaproteobacteria.o__Caulobacterales	LR	0.844786137
511	k__Bacteria.p__Proteobacteria.c__Alphaproteobacteria.o__Sphingomonadales.f__Erythrobacteraceae.g__Erythrobacter	LR	0.837153416
512	k__Bacteria.p__Proteobacteria.c__Epsilonproteobacteria	LR	0.486861099
513	k__Bacteria.p__Actinobacteria.c__Actinobacteria.o__Actinomycetales.f__Mycobacteriaceae	LR	0.157247068
514	k__Bacteria.p__Actinobacteria.c__Actinobacteria.o__Actinomycetales	LR	0.215863602
515	k__Bacteria.p__Bacteroidetes.c__Bacteroidia.o__Bacteroidales.f__Porphyromonadaceae.g__Proteiniphilum	NC	0.147299139
516	k__Bacteria.p__Chloroflexi	NC	0.571581844
517	k__Bacteria.p__Deinococcus_Thermus.c__Deinococci.o__Thermales.f__Thermaceae	LR	0.523616008
518	k__Bacteria.p__Proteobacteria.c__Gammaproteobacteria.o__Cardiobacteriales.f__Cardiobacteriaceae	NC	0.571581844
519	k__Bacteria.p__Proteobacteria.c__Gammaproteobacteria.o__Legionellales.f__Coxiellaceae	NC	0.147299139
520	k__Bacteria.p__Firmicutes.c__Bacilli.o__Lactobacillales.f__Aerococcaceae	LR	0.904537352
521	k__Bacteria.p__Proteobacteria.c__Betaproteobacteria.o__Hydrogenophilales.f__Hydrogenophilaceae.g__Petrobacter	LR	0.605160257
522	k__Bacteria.p__Actinobacteria.c__Actinobacteria.o__Coriobacteriales.f__Coriobacteriaceae.g__Olsenella	NC	0.929815628
523	k__Bacteria.p__Firmicutes.c__Clostridia.o__Clostridiales.f__Ruminococcaceae.g__Saccharofermentans	LR	0.003162779
